# Fasting before elective abdominal ultrasound: a systematic review and meta-analysis

**DOI:** 10.1007/s11739-026-04511-x

**Published:** 2026-09-09

**Authors:** Trixi Braasch, Ildiko Hoffmann, Andreas Deckert, Olaf Horstick, Britta Siegmund

**Affiliations:** 1 Department of Gastroenterology, Infectious Diseases and Rheumatology, Charité - University Medical Center Berlin, corporate member of Freie Universität Berlin and Humboldt-Universität zu Berlin, Berlin, Germany; 2 Department of Internal Medicine, Bundeswehr Hospital, Berlin, Germany; 3 Bundeswehr Medical Care Center, Frankenberg, Germany; 4 Injury Epidemiology and Prevention (IEP) Research Group, Turku University Hospital and University of Turku, Turku, Finland; 5 Heidelberg Institute of Global Health (HIGH), Faculty of Medicine and University Hospital, Heidelberg University, Heidelberg, Germany; 6 Cluster of Excellence ImmunoPreCept, Charité - University Medical Center Berlin, corporate member of Freie Universität Berlin and Humboldt-Universität zu Berlin, Berlin, Germany; 7 Charité – University Medical Center Berlin, corporate member of Freie Universität Berlin and Humboldt-Universität zu Berlin, Klinik für Gastroenterologie, CC13 (Campus Benjamin Franklin), Hindenburgdamm 30, 12203 Berlin, Germany

**Keywords:** Abdominal ultrasound, Fasting, Preparation, Imaging, Diagnostic sufficiency

## Abstract

Fasting before elective abdominal ultrasound is widely recommended to improve image quality or visualization of abdominal structures although supporting evidence is limited. We examined whether fasting is associated with improved diagnostic or image quality-related outcomes in adult abdominal ultrasound. We conducted a systematic review and meta-analysis (PROSPERO CRD42024568514) of studies comparing fasting and non-fasting conditions in adults undergoing elective abdominal ultrasound (search date July 18, 2025; 5 databases). Study quality and risk of bias were assessed, and randomized trials were pooled using random effects models. Real-world preparation recommendations were evaluated across international ultrasound society guidelines and patient-facing sources. 14 studies (9 randomized trials, 5 observational studies; 2,208 participants) met the inclusion criteria. Five randomized trials were eligible for meta-analysis; three were included in the primary analysis (*n* = 378). Two trials contributed organ-specific outcomes to sensitivity analyses. Fasting did not improve overall diagnostic sufficiency, defined as adequate visualization to answer the clinical question without repeat imaging (OR 0.97, 95% CI 0.51–1.86, *p* = 0.92; *I*^2^ = 0%). Significant effects were limited to gallbladder visibility and detectability of the common bile duct, with no consistent benefit otherwise. Risk of bias was moderate and overall certainty of evidence was low. Real-world fasting instructions were highly variable and rarely evidence-based. Fasting before elective abdominal ultrasound does not improve diagnostic sufficiency. Observed benefits were limited to the biliary tract. Given the low certainty of evidence and limited observed benefits beyond the biliary tract, strict and prolonged fasting recommendations should be reconsidered.

**Supplementary Information:**

The online version contains supplementary material available at https://doi.org/10.1007/s11739-026-04511-x.

## Introduction

Abdominal ultrasound is a widely used non-invasive imaging technique for assessing a broad range of conditions, yet preparation practices vary widely. Patients are often instructed to fast before the examination, based on the assumption that fasting improves organ visibility through reduction of intestinal gas and prevention of gallbladder contraction [57]. However, the empirical basis for routine fasting before abdominal ultrasound is limited [36]. Point-of-care ultrasound (POCUS) has been demonstrated to be a feasible diagnostic modality that can be performed without the need for fasting [11, 34, 43, 65]. For vulnerable or hospitalized patients, prolonged fasting, in clinical reality often exceeding 12 h [17, 28, 29, 54], can be burdensome and interfere with nutritional therapy [3, 4, 6, 20, 41, 45, 48]. Other procedural disciplines permit clear fluids or light meals prior to a number of diagnostic examinations [1, 18, 35]. Nevertheless, recommendations for fasting persist despite the uncertain diagnostic value for abdominal ultrasound [17, 33, 49, 53, 59]. Whether fasting improves clinically relevant diagnostic outcomes, particularly diagnostic sufficiency, rather than image quality alone, remains uncertain.

This systematic review synthesizes evidence comparing fasting and non-fasting preparation for abdominal ultrasound in adults and reviews current professional and patient-facing recommendations. The aim was to assess whether fasting is supported by the available evidence and to inform more patient-centered, evidence-based ultrasound preparation protocols.

## Materials and methods

### Data sources and searches

This systematic review follows the PRISMA 2020 [38] guidelines and was assessed using AMSTAR 2 [50]. We searched MEDLINE, Embase, CINAHL, Web of Science, and the Cochrane Library from inception to July 18, 2025, using the Ovid, EBSCOhost, Clarivate, and Wiley platforms, respectively. The search strategy combined MeSH terms and free-text keywords related to ultrasound, abdomen, fasting or eating, and image quality or visibility; the MEDLINE strategy is shown in Supplement S1. No language or other search filters were applied. Reference lists of two conference abstract reviews [30, 44] were screened manually, and backward citation searching of included studies was conducted using the online tool citationchaser [22]. The review protocol was registered in PROSPERO (CRD42024568514).

### Study selection

Eligibility criteria followed a predefined PICO framework (Supplement S1). We included studies investigating intra-abdominal ultrasound that reported outcomes on patient preparation, such as fasting or recent food intake and evaluated organ visibility, image quality, procedural feasibility, or the need for repeat examinations. Studies focusing on pediatric populations, emergency ultrasound, patients with complex post-surgical abdomen, or ultrasound techniques not directed at intra-abdominal structures were excluded. Studies on intra-abdominal elastography, gastric emptying, or abdominal fat layers were also excluded because these do not assess intra-abdominal organ visibility. Eligible designs included randomized controlled trials (RCTs) and observational studies. Case reports, reviews, and gray literature were excluded. For studies available only as abstracts, corresponding authors were contacted to determine whether a peer-reviewed full-text publication existed; if not, the study was excluded.

**Table 1:** Characteristics of included studies

Study (Author, Year)	Design	Country	Population, Setting	*N*	Groups	Outcome relevant for the review	Main result
Goldschmidt (2021)*^a^	RCT, 3-arm	USA	Outpatients; abdominal aorta	90	Fasting 8–12 h (NPO) vs Clear liquids vs No restrictions	Sufficiency of visualization (sufficient vs. limited) and sufficiency for confident diagnosis	No statistical difference in visualization in the three arms. Confidence of diagnosis was 100%
Sinan (2003)*	RCT, 2-arm	Kuwait	Outpatients; abdominal US	104	Fasting >6 h till 08:00–11:00 h ±water (500 ml) vs Normal breakfast ±water (500 ml)	Visibility rating (3-graded: excellent, fair vs. unacceptable), gas amount	Comparable visibility and bowel gas levels between fasting and breakfast groups
Ehrenstein (2009)*	RCT, 2-arm	Germany	Inpatients, general medicine ward; abdominal US	130	Fasting overnight till 9:00–14:00 h vs Breakfast	Imaging index (3-graded: 0, 0.5 and 1 point), need for repeat scan	No significant difference in image indices or repeat scans between fasting and non-fasting
Schreiner (2025)*^b^	RCT, 3-arm, subgrouped	Germany	Inpatients, internal medicine, neurology, otolaryngology; abdominal US	215	Fasting vs Breakfast vs Oral supplement (100 or 300 kcal)	Organ visibility (graded by 1–5 Likert-scale), need for repeat scan; subgroups by examiner experience	Comparable visibility and repeat scan rates across all three arms Only meaningful difference for inexperienced examiners in CHD with liquid preparation
Windler (1985)*	RCT, 2-arm	Germany	Inpatients + emergency room patients; abdominal US	226	Fasting overnight or >4 h vs 6 meals/day	Organ visibility graded 1–3 (only biliary tract data available)	No consistent diagnostic difference due to fasting Slightly poorer biliary visibility in non-fasting (grade 1=bad: 9% vs 5%, grade 3=excellent: 63% vs 73%)
Sisler (1995)	RCT; within-subject for ultrasound, crossover	Canada	Healthy volunteers; GI tract	24	Fasting vs Cellulose ingestion (900 ml)	Subjective visibility (0–4)	Better visibility scores after cellulose compared to fasting; statistically significant, but not clinically significant
Pinto (2011)	RCT, 3-arm	Brazil	Outpatients, mixed diagnoses; Abdominal US	609	Fasting >6 h + water (800 ml) vs + antiflatulent vs + laxative	Visibility score (0–6)	No difference in examination quality was seen among patients with or without medication Other variables, such as obesity and increased abdominal wall thickness, also impacted the image quality
Samovsky (2001)	Prospective, observational; within-subject for ultrasound	Israel	Patients with recurrent RUQ pain; Gallbladder, CBD	5	Fasting ~12 h overnight vs 30 min after fatty meal (one glass of milk = 170 kcal or 50 g chocolate = 270 kcal)	Gallbladder dilation, CBD visibility	Fatty meal improved gallbladder visibility in 5 patients
Alihosseini (2024)	Case–control; within-subject for ultrasound	Iran	IBS patients + healthy controls; Gallbladder, cystic artery and SMA	34	Fasting ~12 h vs Chocolate (approx. 540 kcal)	SMA visibility through SMA duplex	SMA assessable in all; physiological effects seen, but no diagnostic outcome assessed
Hedayati (2011)	Observational	USA	Mixed (in/outpatients); renal artery duplex	250	Fasting (> 4 h) vs Non-fasting	3-graded: diagnostic/limited/non-diagnostic scan rate	No association between fasting status and scan quality. 35% exams incomplete
Tous (1997)	Diagnostic accuracy; within-subject for ultrasound	Spain	Referred after endoscopy with gastric pathology; Gastric US	413	Fasting vs Water (400 ml)	Visibility of stomach and stomach pathology, sensitivity/specificity	Water ingestion aided stomach detection
Fisher (2004)	RCT (randomized crossover trial) for tegaserod Observational; within-subject for ultrasound	USA	IBS patients in drug trial for tegaserod; Gallbladder, CBD/CHD	53	Fasting (> 8 h, starting 21:00 h) vs liquid test meal (360 kcal)	visualization failure of CBD/CHD was reported	Overall failure rate for biliary visualization: 5.8% (distal duct), 2,6% (mid duct), 0,42% (proximal duct), 0,18% (all three levels at once); calculation from predominantly postprandial exams (82%)
Takizawa (2015)	Prospective, observational; within-subject for ultrasound	Japan	Healthy volunteers; US of liver, gallbladder, pancreas, kidney, spleen	9	Fasting (> 8 h) vs oral supplement (200 kcal)	subjective image quality (good/fair/ poor), gallbladder long axis and area	All organs rated ‘Good’ after 3 h post-ingestion, <3 h pancreas and gallbladder were poor
Breyer^c^ (2010)	RCT (randomized crossover trial, fasting-postprandial) with small fasting–fasting control group	USA	Healthy volunteers; Gallbladder	46^c^	Fasting (≥ 8 h) vs 30 min post-fatty meal (480–790 kcal, 3–51 g fat)	Visibility of gall bladder	Visibility of gallbladder was 100% in both arms Gallbladder area decreased 20% 30 min after food

RCT randomized controlled trial, US ultrasound, SMA superior mesenteric artery, CBD common bile duct, CHD common hepatic duct, IBS irritable bowel syndrome, RUQ right upper quadrant, GI Gastrointestinal, * Study included in meta-analysis, a Included in meta-analysis: Fasting vs. no restrictions, b Included in meta-analysis: Fasting versus breakfast. Experienced examiners only; inexperienced examiners for subgroup analysis, c 59 volunteers enrolled, demographics for enrolled population

### Deduplication, screening, and data extraction

Duplicates were removed using the Deduplicator tool [16]. Two reviewers (TB, IH) independently screened titles and abstracts for eligibility criteria using the Screenatron software [7]. Potentially relevant studies were assessed in full text. One reviewer (TB) extracted data on study characteristics (authors, year, journal, country, design), population (sample size, age, gender, BMI), technical aspects (equipment, operator expertise), and outcomes. A second reviewer (IH) verified the extracted data. Disagreements were resolved by consensus.

### Quality and risk-of-bias assessment

Risk of bias was assessed independently by two reviewers (TB, IH) using the RoB 2 tool for RCTs [56] and ROBINS-I for observational studies [55]. Discrepancies were resolved by consensus. In addition, reporting quality was evaluated using the CONSORT 2010 checklist for RCTs [25] and the STROBE statement for observational studies [62]. We planned to assess publication bias visually using a funnel plot. The certainty of evidence for the primary outcome was evaluated using the GRADE framework [21], considering risk of bias, inconsistency, indirectness, imprecision, and publication bias.

### Data synthesis and analysis

Pre-defined outcomes relating to image evaluability (organ visibility, subjective visibility ratings, procedural feasibility, or the need for repeat examinations) were harmonized into a dichotomous measure of diagnostic sufficiency, defined as adequate visibility to answer the clinical question without repeat imaging. Between-subject RCTs comparing fasting versus non-fasting conditions with dichotomous outcomes of diagnostic sufficiency were pooled. Within-subject trials, although methodologically strong in controlling inter-individual variability, were not included because combined paired and unpaired designs produce non-comparable effect estimates, and a paired-design meta-analysis was not pre-specified for this review. Studies with continuous outcomes or without a suitable comparator were synthesized narratively. Studies with 100% visibility in both groups were excluded due to ceiling effects. Sensitivity analyses addressed risk of bias, examiner experience, and heterogeneity in outcome definitions. Pre-defined subgroup analyses focused on the gallbladder and common bile duct (CBD), with proximal duct segment data prioritized where available; trials reporting composite biliary tract outcomes were included in sensitivity analyses. Evaluability was categorized as lenient (at least satisfactory visibility) or strict (excellent visibility) to accommodate the variation in study-level outcome definitions.

Pooled effect estimates were calculated as odds ratios (OR) with 95% confidence intervals using a random effects model (restricted maximum likelihood). Heterogeneity was assessed using *I*^2^ and τ^2^.

### Assessment of online fasting recommendations

To complement the systematic review, real-world fasting recommendations were assessed descriptively. Patient-facing instructions for elective abdominal ultrasound were evaluated across four sources. All acute care hospitals and out-patient practices in Berlin, Germany, were screened for publicly available preparation instructions (see Methods in Supplement S12). Google searches for “preparation before abdominal ultrasound” were conducted with cleared cache and cookies. The first ten healthcare-related results were analyzed [14]. Artificial intelligence (AI) chatbot responses (Gemini and ChatGPT-5) were retrieved using a similar query. Outputs were analyzed descriptively as examples of publicly accessible AI-generated health information. Recommendations from major international ultrasound societies were also reviewed (Supplement S11). Data were extracted manually and cross-checked. Results were summarized descriptively, including fasting durations and content categories; hospital and out-patient recommendations were compared qualitatively.

## Results

### Study selection

The search yielded 6,496 records. After removing 1,245 duplicates, 5,251 titles and abstracts were screened, of which 25 full texts were assessed. Six conference abstracts were excluded because no full-text was available despite author contact (Supplement S6). Six full-text articles did not meet the eligibility criteria (Supplement S5). 13 studies were included. Backward citation searching identified one additional eligible study, resulting in 14 included studies overall. Details are presented in the PRISMA flow diagram (Supplement S1). One included study [66] comprised both emergency and non-emergency examinations, but these could not be analytically separated. As fasting status was recorded for the entire cohort, this study was retained.

### Characteristics of included studies

14 included studies (published 1985–2025) comprised 9 RCTs [5, 12, 15, 19, 40, 47, 51, 52, 66] and 5 observational studies [2, 23, 46, 58, 60], including a total of 2,208 participants (sample size 5–609). Most studies compared fasting with food or fluid intake and assessed visibility of abdominal organs during elective ultrasound. Seven studies primarily compared fasting with non-fasting in terms of organ visibility during abdominal ultrasound. [5, 12, 19, 47, 51, 58, 66]; the remainder assessed visibility-related measures as a secondary or exploratory outcome. Ultrasound outcomes included subjective visibility ratings, diagnostic sufficiency, or the need for repeat examinations, judged by examiners or blinded reviewers. Six studies [12, 40, 47, 51, 58, 66] evaluated overall abdominal ultrasound, four [2, 5, 15, 46] the gallbladder with or without the common bile duct, including the superior mesenteric artery in one study [2]. One study each assessed the aorta [19], the renal artery duplex [23], and the stomach [60]. Another study [52] examined the upper gastrointestinal tract, including stomach, duodenum, pancreas, gallbladder, and spleen. Examiners were predominantly experienced radiologists or physicians, either single operators or small teams. One study involved emergency room residents only [5]. One study compared experienced and inexperienced internal medicine examiners [47]. Three studies used experienced certified technicians [19, 23, 58]. One study did not report examiner experience but applied a standardized videotaped protocol evaluated by a single expert [15]. Test preparations varied substantially. Five studies used standard solid breakfasts [12, 19, 47, 51, 66] and one used a fast-food meal [5]. Three studies tested water ingestion [19, 40, 60] and one tested cellulose-based fluids [52], with volumes from 400 to 900 ml. Other interventions included oral supplements or liquid meals [47, 58], chocolate [2, 46], and milk [46]. Fasting durations ranged from approximately 4 h to overnight (> 12 h), with some studies applying strict NPO instructions. Study populations included inpatients, outpatients, and healthy volunteers. Mean or median ages ranged from 31.6 to 72.5 years. Among 1,990 participants with reported sex, 1,154 (58%) were female. One study included only women [15], while the others enrolled mixed populations with 41.5–78% females. BMI was reported in 9 studies (means 24.5–29.6 kg/m^2^). One study included only participants with BMI < 30 kg/m^2^ [2], while another excluded those with BMI >34 kg/m^2^ [15].

Main study characteristics are summarized in Table 1; additional demographic and technical information is shown in Supplement S2.

### Findings from included studies

Across all 14 studies, fasting did not consistently improve visibility-related outcomes in elective abdominal ultrasound. Ten studies (71%) found no relevant overall diagnostic or visibility-related difference between fasting and non-fasting conditions, while the remaining studies addressed postprandial physiology or organ-specific outcomes.

Among the five studies directly comparing fasting and non-fasting strategies in abdominal or biliary ultrasound [5, 12, 47, 51, 66], visibility-related outcomes did not differ meaningfully. Four studies evaluated multiple abdominal organs [12, 47, 51, 66] and one focused solely on the gallbladder and biliary tract [5]. Two studies [12, 47] reported no statistical difference in repeat-examination rates. Three studies reported isolated effects: Ehrenstein et al. [12] observed a nominal, non-significant advantage of fasting for gallbladder visibility (73% versus 56%), with a significant effect of fasting limited to a post hoc subgroup of male patients. Windler et al. [66] observed significantly better biliary ratings under fasting conditions, although the absolute difference was small: non-diagnostic examinations decreased from 9 to 5%, while overall image ratings remained similar. Schreiner et al. [47] found a difference only for the common hepatic duct among inexperienced examiners, where liquid oral nutritional supplements outperformed breakfast and fasting.

Two studies assessed postprandial or pharmacologically induced physiological effects on organ visibility using ultrasound as a measurement tool rather than a diagnostic endpoint [15, 58]. Takizawa et al. [58] reported reduced visibility of the pancreas and gallbladder within three hours after a meal, with no differences thereafter; all other intra-abdominal organs had fair or good visibility under all conditions. Three studies evaluated intra-abdominal vessels and found no effect of fasting. Goldschmidt et al. [19] reported similar rates of sufficient versus limited aortic visibility across prolonged fasting, clear liquids, and unrestricted intake, with maintenance of 100% diagnostic certainty irrespective of preparation. Hedayati et al. [23] observed no influence of fasting on the completeness of renal artery duplex examinations. Alihosseini et al. [2] found no relevant fasting-related differences in Doppler assessment of the cystic or superior mesenteric arteries; postprandial SMA flow was obtainable in all participants. Two studies investigated exclusively liquid ingestion. Sisler et al. [52] investigated ingestion of 900 ml of cellulose solution and reported improved visibility of the duodenum, pancreas, and stomach, with qualitative gains for the spleen and left kidney, but no differences between cellulose concentrations. The proximal small bowel visibility showed only limited improvement. Pinto et al. [40] tested ingestion of 800 ml of liquid after more than six hours of fasting, with additional arms receiving dimethicone or dimethicone plus bisacodyl; visibility did not differ between medicated and unmedicated groups. Two additional studies examined targeted postprandial or fluid-based interventions beyond standard fasting versus non-fasting comparisons. Samovsky et al. [46] demonstrated that administration of a fatty stimulus (chocolate or milk) improved gallbladder visibility in patients with contracted gallbladders despite prior fasting. Similarly, Tous et al. [60] found that water ingestion increased stomach detectability, from 64 to 72% in the fasting state to 93–94% following fluid intake, with results varying by the ultrasound probe used.

### Risk of bias and quality assessment

Among the nine RCTs, one study had low risk of bias [12], five moderate risk [15, 19, 40, 47, 51], of which three were affected only in the randomization domain [19, 40, 47], and three high risk [5, 52, 66], due to inadequate randomization, baseline imbalance, and missing outcome data (Supplement S4A). All five non-randomized studies [2, 23, 46, 58, 60] had moderate risk of bias, mainly related to confounding, incomplete baseline data, participant selection, and outcome measurement (Supplement S4B).

Most studies reported no external funding or provided no funding statement. One RCT [19] received support from a nonprofit foundation, and no studies reported commercial or industry sponsorship.

Regarding reporting quality, four RCTs were rated good [12, 19, 40, 47] and five moderate [15, 51, 52, 66] (Supplement S3A). Among non-randomized studies, three were rated good [2, 23, 60] and two moderate [46, 58] (Supplement S3B). One study with declared conflicts of interest [47] was independently reassessed by a third reviewer.

### Meta-analysis

Five RCTs were eligible [12, 19, 47, 51, 66] for meta-analysis based on dichotomous outcomes. An additional RCT [5] also reported a dichotomous outcome but was excluded due to its within-subject design and a ceiling effect. The remaining three RCTs [15, 40, 52] were excluded because of within-subject designs or non-dichotomous outcome reporting (Supplement S7).

Three RCTs [12, 47, 51] contributed to the primary analysis, while two additional studies [19, 66] were included in sensitivity analyses because only aorta or biliary tract outcomes were reported. Organ-focused ultrasound was evaluated using lenient (satisfactory visibility) or strict (excellent visibility) criteria (see Methods), and mirror analyses were conducted using the inexperienced-examiner dataset from Schreiner et al. [47].

### Overall diagnostic sufficiency:

**Fig. 1:**
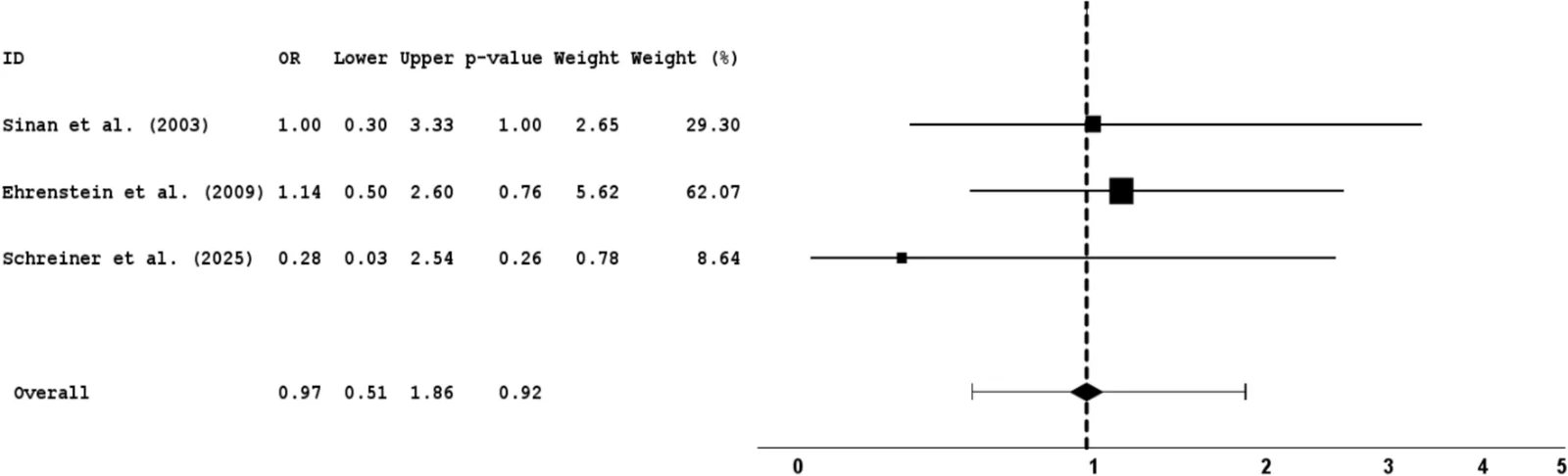
Forest plot of overall abdominal diagnostic sufficiency in fasting versus non-fasting patients

The primary analysis of the three RCTs [12, 47, 51] showed no association between fasting and improved diagnostic outcomes (*n* = 378; pooled OR 0.97, 95% CI 0.51–1.86; *p* = 0.92; *I*^2^ = 0%; Fig. 1). Including the study by Goldschmidt et al., which assessed the aorta only [19], yielded comparable results (*n* = 438; pooled OR 0.83, 95% CI 0.46–1.47; *p* = 0.52; *I*² = 0%).

### Gallbladder evaluability:

In three RCTs [12, 47, 51], fasting was not associated with improved visibility under lenient criteria. Under strict criteria, a significant benefit was observed (*n* = 350; pooled OR 1.89, 95% CI 1.16–3.07; *p* = 0.01; *I*^2^ = 0%).

### CBD evaluability:

In three RCTs [12, 47, 51], fasting was associated with a benefit under lenient criteria (*n* = 378; pooled OR 3.06, 95% CI 1.19–7.86; *p* = 0.02; *I*^2^ = 0%), but not under strict criteria. Using the inexperienced examiner dataset [47] in the mirror analysis reproduced the same pattern, with benefits under lenient criteria only, and consistently lower absolute visibility rates. Windler et al. [66], reporting composite biliary tract outcomes, was included only in sensitivity analyses. It did not make the strict CBD analysis significant in either dataset (experienced: *p*=0.08; mirror: *p*=0.05) and rendered the lenient mirror analysis non-significant.

Pooled estimates of all primary analyses are summarized in Table 2, while full numerical results and trial-level outcome definitions are provided in Supplement S8.

**Table 2:** Meta-analyses of overall diagnostic sufficiency in fasting versus non-fasting patients (REML)

Analysis	*N*	Studies included	Pooled OR (95% CI)	*p*	*I*^2^
Primary	378	3 (Ehrenstein et al., Schreiner et al. [experienced], Sinan et al.)	0.97 (0.51–1.86)	0.92	0%
Sensitivity	438	4 (above + Goldschmidt et al. [aorta only])	0.83 (0.46–1.47)	0.52	0%
Mirror	378	3 (Ehrenstein et al., Schreiner et al. [inexperienced], Sinan et al.)	1.16 (0.65–2.06)	0.61	0%

n number of participants, OR odds ratio, CI confidence interval, *p*-value <0.05 = significant, I^*2*^ heterogeneity, REML restricted maximum likelihood

### Publication bias and certainty of evidence (GRADE)

Publication bias was not assessed with funnel plots because all meta-analyses included fewer than ten studies. The certainty of evidence for diagnostic sufficiency was rated low using GRADE, with downgrades for inconsistency, imprecision, and suspected publication bias. Detailed domain-level assessments are provided in Supplement S9.

### Assessment of current fasting recommendations

Five international professional ultrasound societies provided heterogeneous fasting recommendations (Supplement S11), generally intended to ensure gallbladder distension and reduce bowel gas. Suggested fasting durations ranged from 4 h to 4–6 h, ≥6 h, or overnight, while one did not specify a defined duration and left preparation to the provider. Only one society [36] considered fasting unnecessary for routine intestinal ultrasound but recommended longer fasting for selected indications and was the only society to explicitly support its recommendations with cited literature.

Among 14 healthcare-related websites (10 US, 4 UK), 93% (13/14) recommended fasting, typically for 4–12 h, most commonly 6–8 h. Only 2/14 provided any rationale, and none cited scientific evidence or professional guidelines (Supplement S10). Similarly, both AI-based chatbots assessed (Gemini and ChatGPT-5) recommended fasting before elective abdominal ultrasound, generally advising 6–8 h of food abstinence without referencing supporting evidence. Full transcripts are provided in Supplement S10.

Among 36 acute care hospitals in Berlin, only three (8.3%) provided publicly accessible preparation instructions, all from radiology departments, with recommendations ranging from no fasting to an unspecified “empty stomach” or overnight fasting. Among 75 ambulatory providers, 21 reported explicit preparation instructions, of which 10 (47.6%) recommended prolonged fasting (≥ 8 h or overnight). Detailed results are provided in Supplement S12.

## Discussion

To our knowledge, this is the first systematic review evaluating fasting before routine elective abdominal ultrasound. Across study designs and clinical settings, no consistent or clinically meaningful benefit of fasting was observed for overall diagnostic outcomes. Consistently, the meta-analysis showed no effect on overall diagnostic sufficiency, an outcome reflecting clinical decision-making rather than isolated image quality metrics. Additionally, conference abstracts (Supplement S6) further support that fasting beyond three hours provides no discernible benefit.

Although overall diagnostic sufficiency was unaffected, aggregated analyses may mask outcome-specific definition-dependent effects. In biliary subgroups, effects depended on outcome definitions. For the gallbladder, fasting improved evaluability only under strict criteria, reflecting higher image quality thresholds rather than an increase in evaluable examinations, with limited relevance for routine diagnostics. For the proximal CBD, fasting reduced non-evaluable examinations under lenient criteria but showed no consistent benefit under stricter definitions. Accordingly, fasting appears more relevant for minimizing incomplete CBD assessments than for achieving high-detail visibility. Mirror analyses in inexperienced examiners reproduced similar patterns with lower absolute evaluability.

### Biliary physiological interpretation

The findings support time-limited physiological effects on biliary ultrasound evaluability rather than sustained benefits of prolonged fasting. Transient postprandial changes in gallbladder dimensions occur within three hours after food intake, whereas prolonged fasting does not improve biliary visualization [26, 58, 64]. Adequate gallbladder assessment is feasible in non-fasted individuals [31, 64], and diagnostic agreement for common biliary conditions such as cholelithiasis remains high across fasting states [5, 11]. Variability likely reflects intra- and inter-individual differences in gallbladder contractility related to age, metabolic status, hormonal influences, and medication use [9, 13, 27, 32, 39]. Pediatric data further support that rigid fasting protocols are not required for diagnostically adequate biliary ultrasound examinations [42, 61, 67]. Overall, fasting effects are time-sensitive, and insufficient to justify prolonged or strict fasting in routine biliary ultrasound.

### Fluid intake effects

Fluid intake immediately before imaging may transiently alter abdominal ultrasound conditions by modifying acoustic windows through gastric or intestinal distension and bowel gas displacement, thereby improving visualization of upper abdominal structures [8, 10, 37, 63]. However, across included studies, fluid intake was applied heterogeneously and often at intervals where such effects are unlikely due to gastric emptying [19, 40, 47]. Importantly, the meta-analysis was restricted to preparation conditions reflecting real-life, meal-based intake, excluding isolated liquid or supplement-based interventions [19, 47]. Thus, while acute fluid-induced effects are physiologically plausible, they are not relevant to the preparation strategies analyzed here.

### Real-world context

The analysis of professional society recommendations, healthcare websites, and AI-based tools and search engine-based responses was descriptive and time-bound and should be interpreted as a contextual snapshot. Nevertheless, it provides insights into the current information environment for abdominal ultrasound preparation. Professional societies show heterogeneous fasting recommendations with limited supporting evidence, while public-facing websites and AI tools largely mirror these recommendations without citing primary sources. This persistence of prolonged fasting contrasts with the absence of clinically relevant benefit observed in this review, highlighting a misalignment between evidence and routine preparatory information.

### Clinical implications

Fasting protocols before abdominal ultrasound remain widely applied in routine practice, often without differentiation by diagnostic target or clinical context [17, 33, 53]. In the absence of a demonstrable benefit for overall diagnostic sufficiency, prolonged fasting may expose patients to avoidable risks, particularly in vulnerable populations. Extended fasting, often exceeding 12 h [17, 28, 29, 54], can interfere with nutritional therapy and exacerbate catabolic stress in older patients or chronically ill patients and those at risk of malnutrition [3, 6, 41, 45, 48]. It is also associated with patient discomfort, including hunger, fatigue, anxiety, and nausea [4, 20]. Avoiding unnecessary fasting may represent a modifiable factor in malnutrition prevention and aligns with efforts to reduce non-essential dietary restrictions in clinical care [6, 24]. Accordingly, preparation protocols should prioritize decision-relevant diagnostic adequacy rather than maximal image quality. Simplifying ultrasound preparation strategies may improve patient comfort, reduce logistical burden, and optimize resource use without compromising clinically meaningful diagnostic outcomes.

### Strengths and limitations

This systematic review addresses a clinically relevant and previously insufficiently consolidated question, underlined by the discrepancy between available evidence and routine fasting practice. It followed a rigorous methodological approach in accordance with PRISMA 2020 [38] and AMSTAR 2 [50]. The primary outcome, overall diagnostic sufficiency, was deliberately chosen as a pragmatic and clinically relevant endpoint beyond isolated image quality measures. Subgroup, sensitivity, and mirror analyses supported the robustness of findings across examiner experience, target organs, and outcome definitions. Integration of professional society recommendations and patient-facing online information further enhances the translational relevance.

Limitations include the limited number of studies eligible for meta-analysis, heterogeneous outcome definitions, and inclusion of studies spanning several decades with evolving ultrasound technology, resulting in low certainty of evidence. Although statistical heterogeneity was low, pooled estimates do not imply conceptual homogeneity as outcomes ranged from subjective image quality to functional endpoints such as diagnostic sufficiency or repeat examination. Inherent variability in abdominal ultrasound related to patient characteristics, indication, and examiner experience further limits comparability. While within-subject designs addressed some of this variability, quantitative synthesis was not feasible due to lack of paired data. The geographical distribution of the included studies may limit the generalizability of our findings to populations with different dietary habits and meal composition. Finally, publication bias could not be formally assessed owing to the small number of RCTs, and selective non-publication cannot be excluded.

## Conclusion

Taken together, the available evidence does not support a clinically meaningful benefit of prolonged fasting before routine elective abdominal ultrasound. A pragmatic preparation strategy avoiding rigid fasting requirements appears appropriate, with short or indication-specific fasting reserved for selected biliary assessments where incomplete visualization would have direct clinical consequences.

## Supplementary Information

Below is the link to the electronic supplementary material.

Supplementary file1 (PDF 1631 kb)
