# Supplementary material for: Fasting before elective abdominal ultrasound: a systematic review and meta-analysis
Source: Intern Emerg Med. 2026 Sep 9;21(8):2973–84. doi: 10.1007/s11739-026-04511-x (PMC13751804; doi:10.1007/s11739-026-04511-x)

Fasting before elective abdominal ultrasound: a systematic review and meta-analysis

In: Internal and Emergency Medicine

Trixi Braasch[1,2], Ildiko Hoffmann[3,4], Andreas Deckert[5], Olaf Horstick[5], Britta Siegmund[1,6]

[1] Department of Gastroenterology, Infectious Diseases and Rheumatology, Charité - University Medical Center Berlin, corporate member of Freie Universität Berlin and Humboldt-Universität zu Berlin, Berlin, Germany
[2] Department of Internal Medicine, Bundeswehr Hospital Berlin, Berlin, Germany
[3] Bundeswehr Medical Care Center, Frankenberg, Germany
[4] Injury Epidemiology and Prevention (IEP) Research Group, Turku University Hospital and University of Turku, Finland
[5] Heidelberg Institute of Global Health (HIGH), Faculty of Medicine and University Hospital, Heidelberg University, Heidelberg, Germany.
[6] Cluster of Excellence ImmunoPreCept, Charité - University Medical Center Berlin, corporate member of Freie Universität Berlin and Humboldt-Universität zu Berlin, Berlin, Germany

Corresponding author:
Trixi Braasch, trixi.braasch@charite.de

Supplements

Supplement S1: PRISMA Flowchart, PICO and Search Strategy
Supplement S2: Additional Findings Related to Image Quality or Study Design
Supplement S3: Quality Assessment
Supplement S4: Risk-of-Bias Assessment
Supplement S5: Excluded Full-Text Studies with Reasons
Supplement S6: Conference Abstracts
Supplement S7: Justification for Study Exclusion from Meta-analysis
Supplement S8: Meta-Analysis: Tables and Forest Plots, Including Sensitivity and Subgroup Analyses
Supplement S9: GRADE Summary
Supplement S10: Patient-Facing Online Fasting Instructions for Abdominal Ultrasound (Google, Chatbot GEMINI, Chatbot ChatGPT-5)
Supplement S11: Ultrasound Societies Recommendations
Supplement S12: Web-Based Patient-Facing Preparation Recommendations for Elective Abdominal Ultrasound in Ambulatory Practices and Hospitals in the Federal State of Berlin, Germany

Supplement S1: PRISMA Flowchart, PICO and search strategy

PRISMA Flowchart

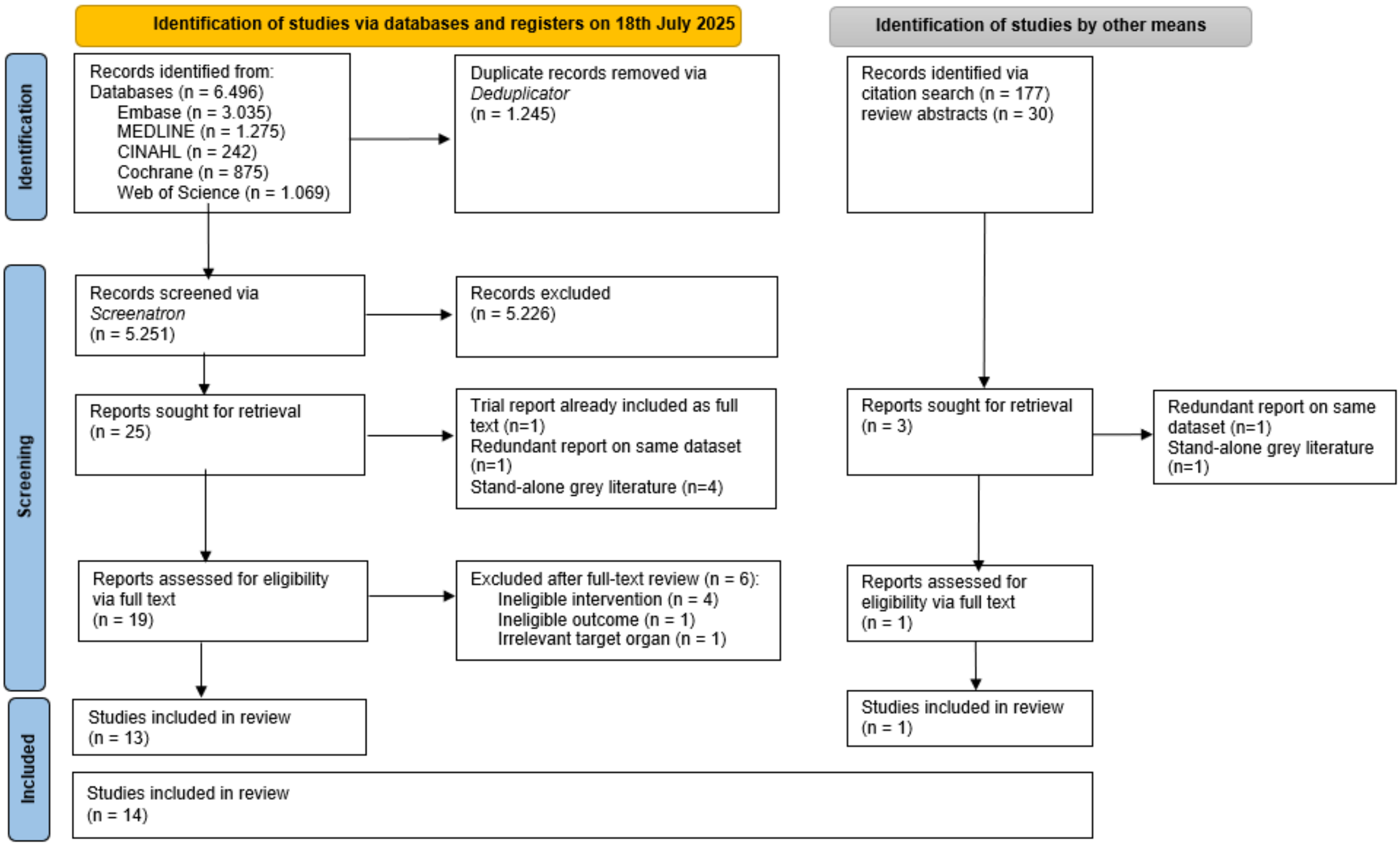

Source: Page MJ, et al. BMJ 2021;372:n71. doi: 10.1136/bmj.n71.

S1 Table 1

<u>PICO- Framework</u>:

Population (P): Adults (≥18 years) undergoing intra-abdominal ultrasound

Intervention (I): Patient preparation involving fasting or controlled food intake

Comparator (C): No specific preparation or alternative preparation protocols

Outcomes (O): Visibility, image quality, feasibility, or need for repeat examination

S1 Table 2: Detailed Search Strategy in Ovid MEDLINE (ALL)

Database: Ovid MEDLINE(R) ALL (1946 to July 17, 2025)
Interface: Ovid
Date last searched: July 18, 2025

#	Search Term	Records
1	Ultrasonography/	209,821
2	ultrasound.ab,ti.	350,147
3	sonograph*.ab,ti.	62,886
4	ultrasono*.ab,ti.	132,822
5	Abdomen/	43,382
6	abdom*.ab,ti.	443,294
7	Fasting/	40,216
8	fasting*.ab,ti.	141,615
9	eat*.ab,ti.	140,797
10	starv*.ab,ti.	45,166
11	food*.ab,ti.	702,872
12	diet*.ab,ti.	755,241
13	nil.ab,ti.	7,787
14	npo.ab,ti.	1,233
15	perform*.ab,ti.	4,842,117
16	visibil*.ab,ti.	22,855
17	qualit*.ab,ti.	2,021,879
18	success*.ab,ti.	1,587,657
19	assess*.ab,ti.	4,466,458
20	imag*.ab,ti.	1,737,321
21	visual*.ab,ti.	855,521
22	1 or 2 or 3 or 4	558,813
23	5 or 6	456,232
24	7 or 8 or 9 or 10 or 11 or 12 or 13 or 14	1,541,944
25	15 or 16 or 17 or 18 or 19 or 20 or 21	11,516,066
26	22 and 23 and 24 and 25	1,275

Supplement 2

S2 Table 1: Additional Findings Related to Image Quality or Study Design Potentially Influencing Outcomes

Study	N	Age (years)	Female (%)	BMI (kg/m²)	Examiner	Transducer
Goldschmidt (2021)*[a]	90	72.47 ± 8.06	n.a.	29.61 ± 5.93	Accredited registered vascular technologists, plus interpreting physicians	2.25-4.0 MHz (Doppler at least 2.25 MHz)
Sinan (2003)*	104	10–84	n.a.	n.a.	Five experienced radiologists	SSA 270 (Toshiba); 3.75 MHz
Ehrenstein (2009)*	130	57	41.5	~26 (25.7 ± 4.5; 26.1 ± 4.9)	Physicians who had received sonography certification	Sonoline Elegra or Acuson Sequoia 512 (Siemens), EUB 8500 (Hitachi) or Logiq 9 (GE); standard convex (3.5-4.0 MHz) and linear (7.0 MHz)
Schreiner (2025)*[b]	215	65 (18–90)	52	~24.5–26.5	Experienced examiners (specialist in internal medicine with >5 years of experience) vs. Inexperienced examiners: (less than 6 months of experience in US, less than 200 exams)	Aplio 500 or Aplio i800 (Canon)
Windler (1985)*	226	18–>70	43.8	n.a.	Specialist (one of the two authors)	Real-time B-Mode gray-scale sector scanner; 4 or 5 MHz
Sisler (1995)	24	18–40	n.a.	n.a.	Radiologists	SSA-270A (Toshiba); 3.5 MHz curved and linear
Pinto (2011)	609	58 ± 14 (18–88)	75.5	25.6 ± 4.5	Board-certified radiologists with over 5 years of experience	HDI 5000 scanner (Philips); curved-array 3.5 to 7.5 MHz
Samovsky (2001)	5	n.a.	n.a.	n.a.	Radiologist	HDI 3000 ultrasound scanner; 2-5 MHz convex-array
Alihosseini (2024)	34	31.6 (18–45)	58.8	<30	Expert radiologist	WS80A (Samsung); convex C2-8A
Hedayati (2011)	250	59.9 ± 17.8	57	n.a.	registered vascular ultrasound technologists	Philips HDI 5000, Philips iU22
Tous (1997)	413	20–92	42.1	n.a.	One radiologist	Siemens Sonoline 2;

						3.5 MHz and 7.5 MHz
Fisher (2004)	53	18–60	100	16–33[c]	Pre-trained standardized protocol, blinded single reviewer of videos	“real-time high-resolution ultrasonography”
Takizawa (2015)	9	31.8 overall;<br>male: 37.4 (21–58);<br>female: 24.8 (20–35)	44.4	n.a.	Clinical laboratory technician, specializing in abdominal ultrasonography	LOGIQ 9 (G);<br>Typ M7C GII 3-8 MHz
Breyer (2010)	46[d]	40.5 ± 10.9	78	28.1 ± 6.5<br>(17.2–57.8)	Emergency medicine residents	SonoSite Titan;<br>60 mm convex probe or 25 mm micro convex<br>and<br>SonoSite MicroMaxx;<br>phased array 17 mm probe

BMI = Body Mass Index, MHz = megahertz, US = ultrasound

* Study included in meta-analysis
[a] *Included in meta-analysis:* Fasting vs. No restrictions.
[b] *Included in meta-analysis:* Fasting versus breakfast. Experienced examiners only; inexperienced examiners for subgroup analysis.
[c] BMI >34 kg/m² was criterion for exclusion
[d] 59 volunteers enrolled, demographics for enrolled population

Supplement S3: Quality Assessment

S3A: CONSORT-30 (1) Quality Assessment of Included Randomized Controlled Trials (RCTs)

	1	2	3	4	5	6	7	8	9	10	11	12	13	14	15	16	17	18	19	20	21	22	23	24	25	26	27	28	29	30	X
Goldschmidt (2021)																															52 %
Ehrenstein (2009)																															65 %
Schreiner (2025)																															67 %
Sinan (2003)																												-			43 %
Windler (1985)																												-			34 %
Sisler (1995)																												-			48 %
Pinto (2011)																															50 %
Fisher (2004)																															43 %
Breyer (2010)																															40%

Green box = 1-point, yellow box = 0.5-point, red box = 0-points, white box = NA, X (%) = number assigned points divided by number eligible fields, rose box = quality in the third quartile

Quality rating:

first quartile	(x ≤ 25%) = low
second quartile	(25% < x ≤ 50%) = moderate
third quartile	(50% < x ≤ 75%) = good
fourth quartile	(75% < x) = high,
	(x > 90 %) = excellent

S3B: STROBE (2) Quality Assessment of Included Non-Randomized Studies

	1	2	3	4	5	6	7	8	9	10	11	12	13	14	15	16	17	18	19	20	21	22	X
Samovsky (2001)	yellow	green	green	red	yellow	yellow	yellow	green	red	red	-	red	-	yellow	yellow	red	-	green	red	yellow	red	red	34 %
Alihosseini (2024)	green	green	green	green	yellow	green	green	green	yellow	red	green	yellow	red	yellow	green	green	green	green	green	green	yellow	red	75 % (rose)
Hedayati (2011)	yellow	green	green	green	green	yellow	green	yellow	yellow	red	yellow	yellow	yellow	yellow	yellow	yellow	yellow	green	green	green	green	red	65 % (rose)
Tous (1997)	green	green	green	yellow	green	yellow	yellow	green	yellow	green	yellow	yellow	yellow	yellow	green	yellow	red	green	red	yellow	red	red	59 % (rose)
Takizawa (2015)	yellow	green	green	yellow	yellow	yellow	yellow	green	red	red	yellow	yellow	red	red	green	green	red	green	red	yellow	red	red	43 %

Green box = 1-point, yellow box = 0.5-point, red box = 0-points, white box = NA, X (%) = number assigned points divided by number eligible fields, rose box = quality in the third quartile

<u>Quality rating:</u>

first quartile	(x ≤ 25%) = low
second quartile	(25% < x ≤ 50%) = moderate
third quartile	(50% < x ≤ 75%) = good
fourth quartile	(75% < x) = high,
	(x > 90 %) = excellent

Supplement S4: Risk-of-bias Assessment

S4A: Visualization (3) of Rob2 (4) Risk-of-bias Assessment of Included Randomized Controlled Trials (RCTs)

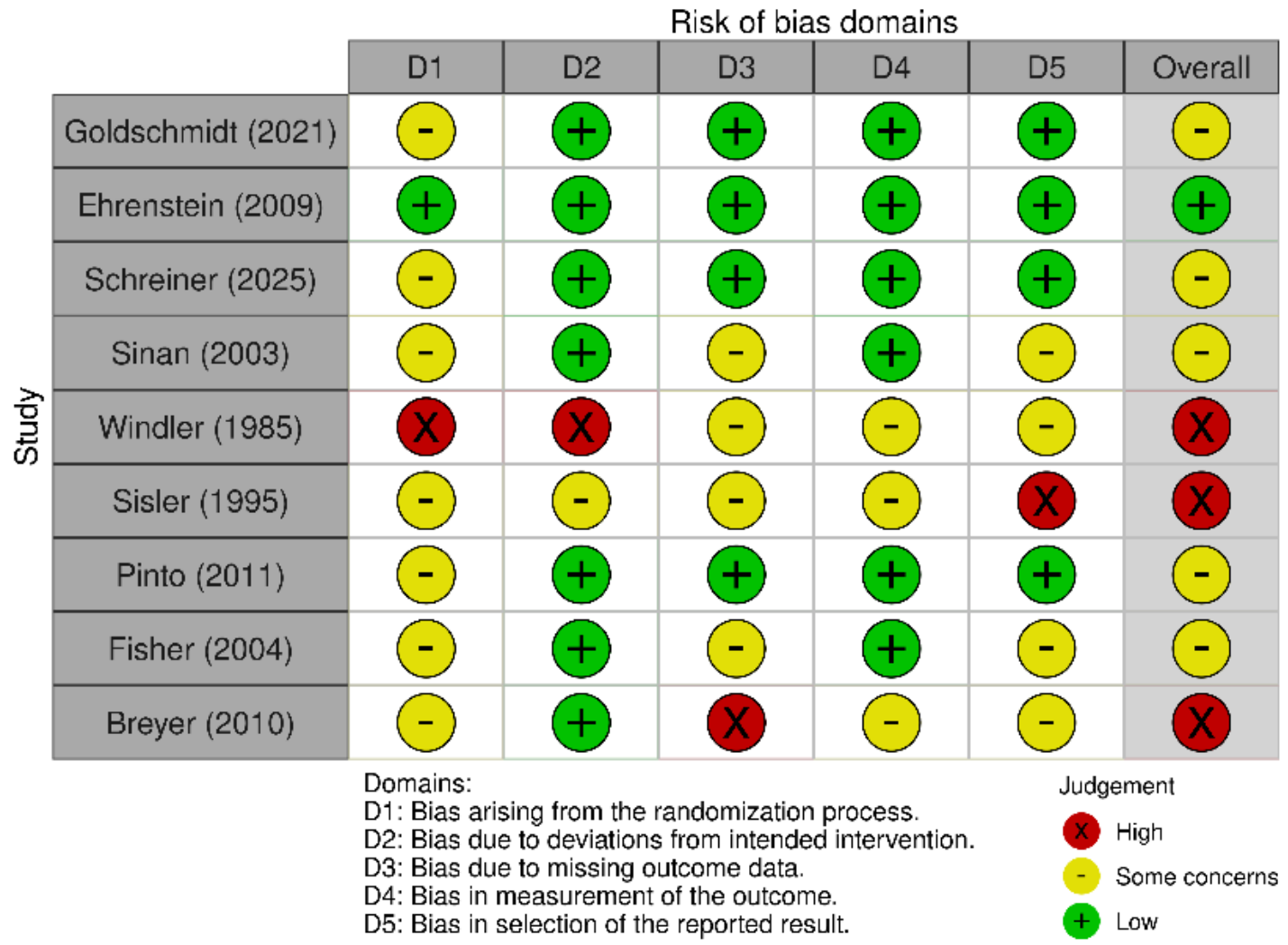

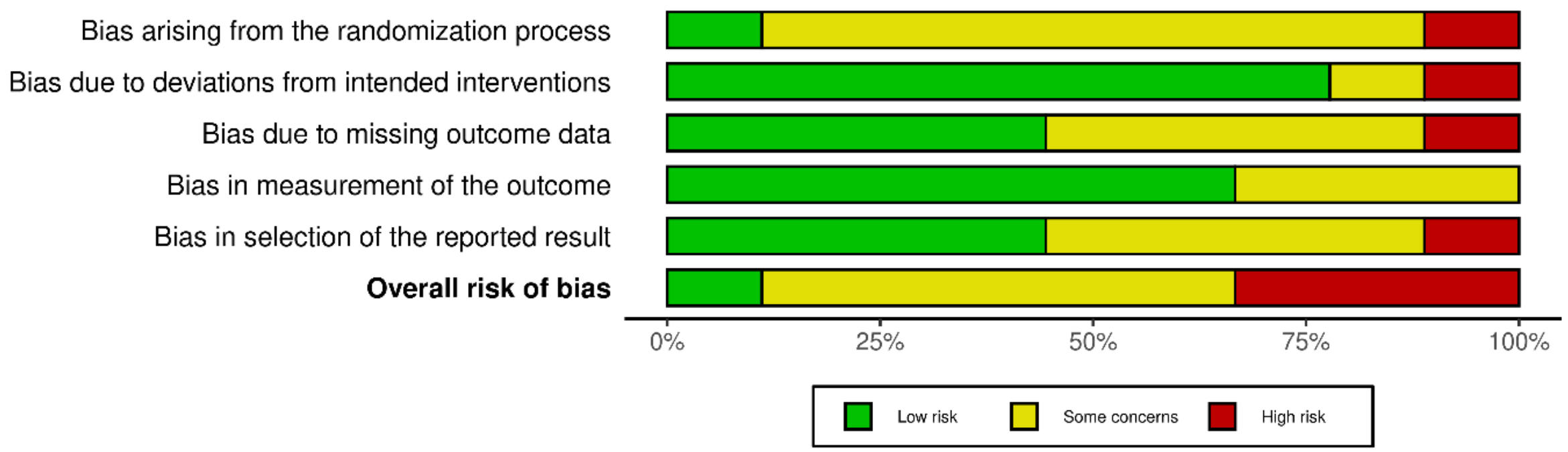

S4B: Visualization (3) of ROBIN-I V2 (5) Risk-of-bias Assessment of Included Non-Randomized Studies

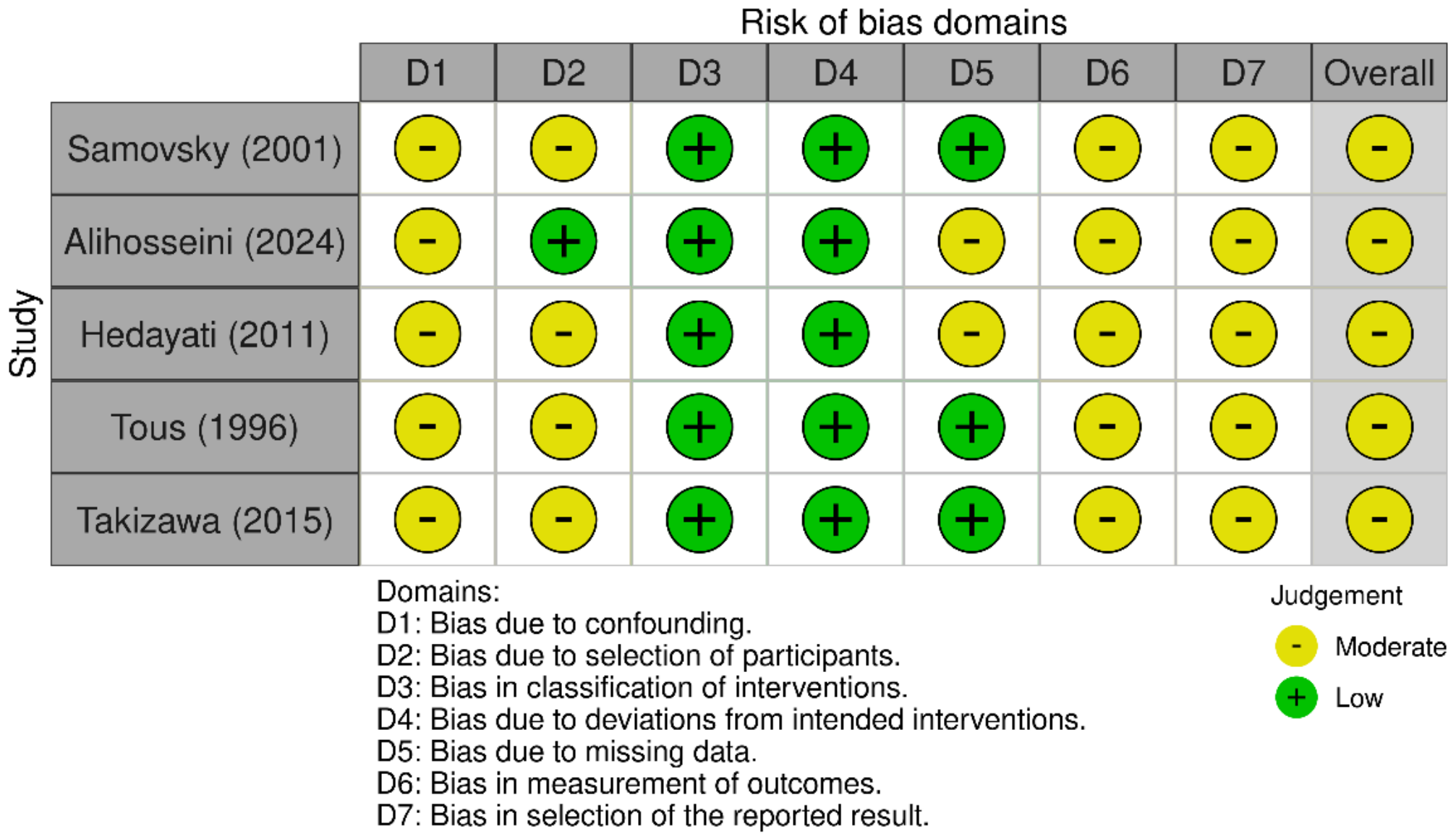

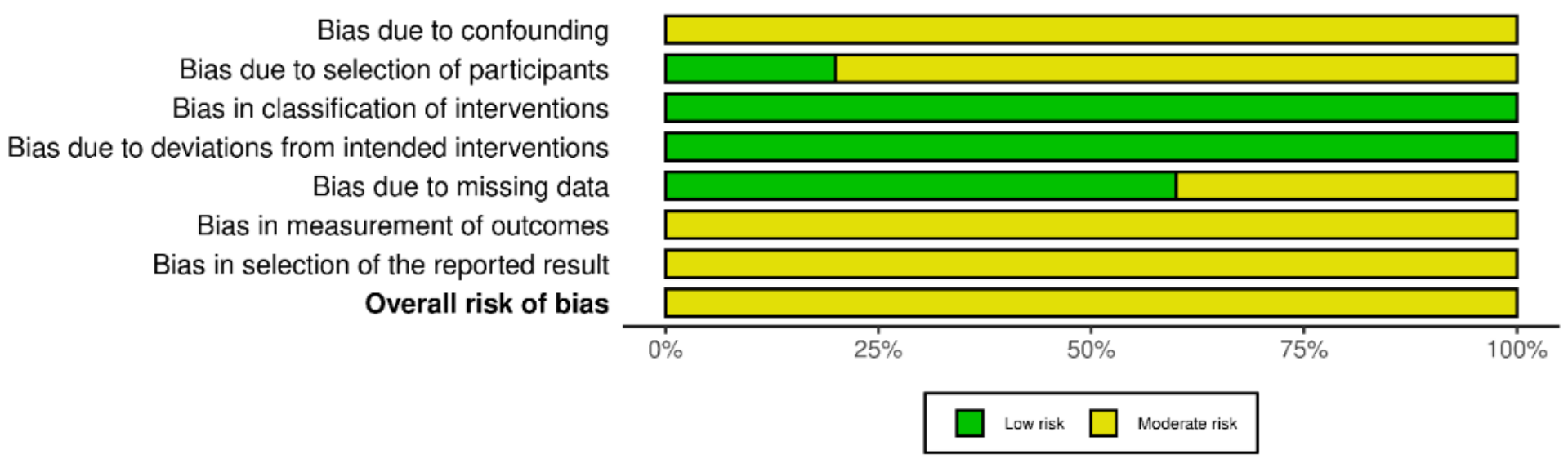

1. Hopewell S, Chan AW, Collins GS, Hróbjartsson A, Moher D, Schulz KF, et al. CONSORT 2025 statement: updated guideline for reporting randomised trials. Bmj. 2025;389:e081123.
2. von Elm E, Altman DG, Egger M, Pocock SJ, Gøtzsche PC, Vandenbroucke JP. Strengthening the Reporting of Observational Studies in Epidemiology (STROBE) statement: guidelines for reporting observational studies. Bmj. 2007;335(7624):806–8.
3. McGuinness LA, Higgins JPT. Risk-of-bias VISualization (robvis): An R package and Shiny web app for visualizing risk-of-bias assessments. Res Synth Methods. 2021;12(1):55–61.
4. Sterne JAC, Savović J, Page MJ, Elbers RG, Blencowe NS, Boutron I, et al. RoB 2: a revised tool for assessing risk of bias in randomised trials. BMJ. 2019;366:l4898.
5. Sterne JA, Hernán MA, Reeves BC, Savović J, Berkman ND, Viswanathan M, et al. ROBINS-I: a tool for assessing risk of bias in non-randomised studies of interventions. BMJ. 2016;355:i4919.

Supplement S5: Excluded Full-Text Studies with Reasons

Article	Reason for exclusion	Short description
Bazzocchi A, et al. The role of ultrasonography in the evaluation of abdominal fat: analysis of technical and methodological issues. Academic Radiology. 2013;20(10). (1)	Different organ, different outcome	Focus on ultrasound-based adiposity measurement (visceral and subcutaneous fat thickness)
Muller AF. Role of duplex Doppler ultrasound in the assessment of patients with postprandial abdominal pain. Gut. 1992;33(4). (2)	Different outcome, different intervention.	Focus on mesenteric blood flow; does not assess impact of fasting on organ visibility
Nilsson A and Forsberg L. The effect of laxatives and enemas on the sonographic visibility of the pancreas. European Journal of Radiology. 1990;10(2). (3)	Different intervention	All groups were fasting and no non-fasting comparator group was included
Philipsen A, et al. Reproducibility of ultrasonography for assessing abdominal fat distribution in a population at high risk of diabetes. Nutrition & Diabetes. 2013;3. (4)	Different organ	Focus on abdominal fat distribution, does not assess the impact of fasting or food intake
Vogel HJP, et al. Abdominal ultrasonography: Improved image quality with the combined use of a diet and laxatives. Journal of Clinical Ultrasound. 1990;18(8). (5)	Different intervention	No group underwent ultrasound in a true postprandial (fed) state. Both groups fasted since the night before exam
Warren PS, et al. The liquid-filled stomach-an ultrasonic window to the upper abdomen. Journal of Clinical Ultrasound. 1978;6(5). (6)	Different intervention No numbers, no comparison.	Lack of comparator group and absence of outcome related to diagnostic visibility or image quality under fasting vs. non-fasting conditions.

1. Bazzocchi A, Filonzi G, Ponti F, Amadori M, Sassi C, Salizzoni E, et al. The role of ultrasonography in the evaluation of abdominal fat: analysis of technical and methodological issues. Academic Radiology. 2013;20(10).
2. Muller AF. Role of duplex Doppler ultrasound in the assessment of patients with postprandial abdominal pain. Gut. 1992;33(4).
3. Nilsson A, Forsberg L. The effect of laxatives and enemas on the sonographic visibility of the pancreas. European Journal of Radiology. 1990;10(2).
4. Philipsen A, Carstensen B, Sandbaek A, Almdal TP, Johansen NB, Jorgensen ME, et al. Reproducibility of ultrasonography for assessing abdominal fat distribution in a population at high risk of diabetes. Nutrition & Diabetes. 2013;3.
5. Vogel HJ, Schipper J, Hermans J. Abdominal ultrasonography: improved image quality with the combined use of a diet and laxatives. Journal of Clinical Ultrasound. 1990;18(8).
6. Warren PS, Garrett WJ, Kossoff G. The liquid-filled stomach--an ultrasonic window to the upper abdomen. Journal of Clinical Ultrasound. 1978;6(5).

Supplement S6: Conference Abstracts (standalone data sets)

Table S6 Conference Abstracts (standalone data sets) Potentially Eligible During Screening

Article	Details	Description	Results
Abbas J et al. Fasting Before Duplex Ultrasound Scan of Abdominal Aorta Is Unnecessary: Comparison of the Results of a Pilot Randomized Blind Study and a Retrospective Cohort. Journal of Vascular Surgery. 2019;70(3). (1)<br><br>Mason J et al. PC008. Fasting Before Duplex Ultrasound Scan of Abdominal Aorta Is Unnecessary: Comparison of the Results of a Pilot Randomized Blind Study and a Retrospective Cohort. Journal of Vascular Surgery. 2019;69(6). (2)	Abstract from the 2019 Midwestern Vascular Surgical Society Annual Meeting<br><br>Same data as Mason et al.<br><br>Senior author contacted: no answer.	pilot RCT (n = 23)<br><br>and a retrospective cohort (n = 3,491)<br><br>Fasting vs. clear liquids vs. regular meal	Non-fasting does not impair image quality before abdominal aortic ultrasound.
Kang D. Is 6 hours of fasting necessary? The effect on image assessability and dilation of the gallbladder with 3 hours fasting prior to an abdominal sonography examination. Sonography. 2021;8(SUPPL 1). (3)	Abstract from ASA2021 Brisbane Hybrid, 27th Annual International Conference of the Australasian Sonographers Association, 28–30 May 2021<br><br>Author contacted via LinkedIn: no answer.	Within-subject observational study (n = 25, healthy adults)	Overall gallbladder visibility did not differ significantly.
Wolstenhulme S, Consiglio H. Ultrasound imaging of the right-side upper abdomen: Revisiting lying compared to standing erect with gastric filling! Ultrasound. 2020;28(2). (4)	Poster Abstract from the 51st Annual Scientific Meeting of the British Medical Ultrasound Society, 10–12 December 2019, Harrogate Convention Centre, Harrogate, UK<br><br>Lead author and colleagues contacted: Retired author, no follow-up address.	observational study (pro/retro-spective?)<br><br>(n= 400)<br><br>fasted vs. non-fasted	Fasting did not significantly improve diagnostic outcomes or organ visibility in routine abdominal ultrasound exams.

Prancisca GN, Rianto S, Wibowo GM. Effect of oral activated charcoal on the performance image visualization upper abdominal ultrasonography. Presented at: 22nd International Society of Radiographers and Radiological Technologists (ISRRT) World Congress Conference; 2022 Dec 15–17; Bangkok, Thailand. J Med Imaging Radiat Sci. 2022;53. (5)	Abstract from the 22nd ISRRT World Congress (Dec 15–17, 2022, Bangkok, Thailand).<br><br>Lead author contacted, no response.	Experimental study with posttest control group design. 30 healthy volunteers underwent upper abdominal ultrasound after either activated charcoal or simethicone. Comparison focused on visualization of stomach, intestine, liver, pancreas, and gallbladder.	No statistically significant difference in organ visibility between activated charcoal and simethicone. Both reduced bowel gas; charcoal may serve as oral contrast alternative.
Westerway SC, Gibson V. A new approach to gallbladder imaging. Ultrasound in Medicine and Biology 2003; 29: S172. (6)<br><br>Westerway S. Ultrasound of the gallbladder – is it necessary to fast? Educational Supplement. Soundeffects. 2003;3:20-22. (7)	Abstract from the 10th WFUMB Congress (Jun 1-4, 2003, Montreal, Canada).<br><br>Later presented as a non-peer-reviewed educational article.	WFUMB 2003 abstract (n=15 volunteers) and<br><br>subsequent non-peer-reviewed educational supplement (n=15 volunteers + 178 routine practice scans)<br><br>nil per mouth vs. light meal vs. high-fat meal.	light non-fat meals did not impair gallbladder/CBD imaging, fatty meals caused full contraction (~2–2.5h)<br><br>52–53% suboptimal visualization in both groups, no significant difference by fasting status.

1. Abbas J, Mason J, Russell T, Kasper G, Paolini D, Acino R, et al. Fasting Before Duplex Ultrasound Scan of Abdominal Aorta Is Unnecessary: Comparison of the Results of a Pilot Randomized Blind Study and a Retrospective Cohort. Journal of Vascular Surgery. 2019;70(3).
2. Mason J, Russell T, Paolini D, Acino R, Lurie F. PC008. Fasting Before Duplex Ultrasound Scan of Abdominal Aorta Is Unnecessary: Comparison of the Results of a Pilot Randomized Blind Study and a Retrospective Cohort. Journal of Vascular Surgery. 2019;69(6).
3. Kang D. Is 6 hours of fasting necessary? The effect on image assessability and dilation of the gallbladder with 3 hours fasting prior to an abdominal sonography examination. Sonography. 2021;8(SUPPL 1).
4. Wolstenhulme S, Consiglio H. Ultrasound imaging of the right-side upper abdomen: Revisiting lying compared to standing erect with gastric filling! Ultrasound. 2020;28(2).
5. Prancisca GN, Rianto S, Wibowo GM. EFFECT OF ORAL ACTIVATED CHARCOAL ON THE PERFORMANCE IMAGE VISUALIZATION UPPER ABDOMINAL ULTRASONOGRAPHY...22nd International Society of Radiographers and Radiological Technologists (ISRRT) World Congress Conference, December 15-17, 2022, Bangkok, Thailand. Journal of Medical Imaging & Radiation Sciences. 2022;53.
6. Westerway SC, Gibson V. A new approach to gallbladder imaging. Ultrasound in Medicine and Biology. 2003;29(5):S172.
7. Westerway SC. Ultrasound of the gallbladder - is it necessary to fast. Soundeffects. 2003;3:20–2.

Supplement S7: Justification for Study Exclusion from Meta-Analysis

Table S7: Studies Included in the Systematic Review but Excluded from Meta-Analysis, with Justification

Study (Author, Year)	Reason for Exclusion from Meta-analysis
Alihosseini (2024)	Within-subject case-control design with a physiological focus. Assessed SMA visibility via Doppler and volume-based measures, not standard B-mode image quality. Indirect visibility of SMA was not a primary or secondary outcome, was deducted. No generalizable diagnostic outcome. High risk of bias due to missing data.
Hedayati (2011)	Observational study without disaggregated group data. Image quality outcome not reported by fasting status. Only reports a non-significant association ($p = 0.155$). High risk of bias due to missing data.
Tous (1997)	Within-subject design evaluating gastroduodenal tumors. No diagnostic imaging quality outcome reported. Comparator was fasting vs. water ingestion, not a generalizable intervention.
Samovsky (2001)	Within-subject design with functional focus on gallbladder volume post-fatty meal in gallstone patients. Did not assess diagnostic sufficiency or general image quality.
Sisler (1995)	Data incomplete or not extractable. Assessed cellulose vs. fasting in healthy volunteers. No binary outcome data available. High risk of bias due to missing data.
Pinto (2011)	Used non-standard comparators (e.g., laxatives, antiflatulents) and only reported mean visibility scores. No binary outcomes extractable.
Fisher (2004)	No stratification of outcomes by fasting status; failure rates reported as aggregate across all timepoints (predominantly postprandial).
Takizawa (2015)	Only qualitative visibility outcomes reported (no numerical proportions per category). No group-level comparison or stratified data (e.g., fasting vs. non-fasting).
Breyer (2010)	Within-subject crossover design with small fasting–fasting control subgroup. Gallbladder visibility was 100% under both fasting and post-prandial conditions, producing a ceiling effect and no informative between-group comparison.

Supplement S8: Meta-analysis Results with Tables and Forest Plots, including Sensitivity and Subgroup Analyses.

S8a. Overall Diagnostic Sufficiency

- Table S8a1. Pooled results of overall diagnostic sufficiency (primary analysis, sensitivity with Goldschmidt, mirror with inexperienced examiners).
- Figure S8a1. Forest plot of overall diagnostic sufficiency (primary analysis).
- Figure S8a2. Forest plot of overall diagnostic sufficiency (sensitivity with Goldschmidt).
- Figure S8a3. Forest plot of overall diagnostic sufficiency (mirror primary analysis with inexperienced examiners).

S8b. Gallbladder Evaluability

- Table S8b1. Pooled results of gallbladder evaluability under lenient and strict definitions.
- Figure S8b1. Forest plots of gallbladder evaluability under lenient definition with mirror analysis.
  - (A) Experienced examiners
  - (B) Mirror: Inexperienced examiners.
- Figure S8b2. Forest plots of gallbladder evaluability under strict definition with mirror analysis.
  - (A) Experienced examiners
  - (B) Mirror: Inexperienced examiners.
- Figure S8b3. Forest plots of gallbladder evaluability - sensitivity analyses (lenient definition).
  - (A) Experienced examiners
  - (B) Mirror: Inexperienced examiners.

S8c. Common Bile Duct (CBD) Evaluability

- Table S8c1. Pooled results of CBD evaluability under lenient and strict definitions.
- Figure S8c1. Forest plot of CBD evaluability under lenient definition.
  - (A) Experienced examiners
  - (B) Mirror: Inexperienced examiners.
- Figure S8c2. Forest plot of CBD evaluability under strict definition.
  - (A) Experienced examiners
  - (B) Mirror: Inexperienced examiners.
- Figure S8c3. Forest plots of CBD evaluability (sensitivity including Windler).
  - (A) Experienced examiners, lenient definition.
  - (B) Mirror: Inexperienced examiners, lenient definition.
  - (C) Experienced examiners, strict definition.
  - (D) Mirror: Inexperienced examiners, strict definition

All meta-analyses were performed using a random-effects model (REML).

Because of the small number of studies and the REML approach, heterogeneity estimates should be interpreted with caution.

Odds ratios (OR) and 95% confidence intervals (CI) are displayed on a logarithmic scale.

S8a. Overall Diagnostic Sufficiency

Definition:

**Study**	**Outcome**
Sinan 2003	Overall unacceptable visualization versus excellent/acceptable
Ehrenstein 2009	hypothetical repeat-evaluation under fasting conditions: yes/no
Schreiner 2025 (exp./inexp.)	Exam diagnostic for clinical indication: yes/no
Goldschmidt 2021	Sufficient versus limited visualization

Table S8a1. Pooled results of overall diagnostic sufficiency
This table is identical to Table 2 in the main manuscript and included here for completeness within the Supplement 8 series.

**Analysis**	**N**	**Studies included**	**Pooled OR (95% CI)**	**p**	**I²**
Primary	378	3 (Ehrenstein 2009, Schreiner 2025 [experienced], Sinan 2003)	0.97 (0.51–1.86)	0.92	0%
Sensitivity	438	4 (above + Goldschmidt 2021 [aorta only])	0.83 (0.46–1.47)	0.52	0%
Mirror	378	3 (Ehrenstein 2009, Schreiner 2025 [inexperienced], Sinan 2003)	1.16 (0.65–2.06)	0.61	0%

Abbreviations: n = number of participants; OR = odds ratio; CI = confidence interval; p-value <0.05 = significant, I^2 = heterogeneity

Figure S8a1. Forest plot of overall diagnostic sufficiency (primary analysis).
This Figure is identical to Figure 1 in the main manuscript; included here for completeness within Supplement 8 series.

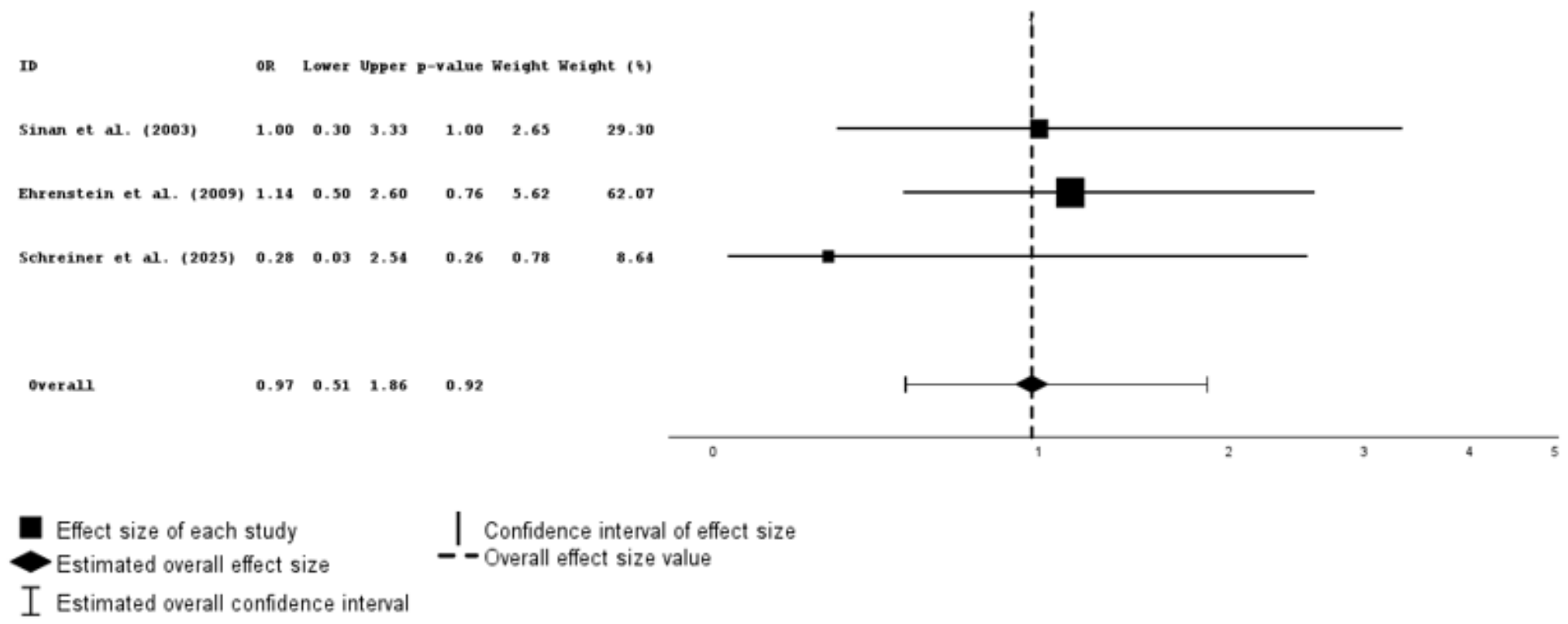

**Study (Year)**	**Fasting (n/N)**	**Non-fasting (n/N)**	**OR (95% CI)**	**p-value**
Sinan (2003)	46/52	46/52	1.00 (0.30–3.33)	1.00
Ehrenstein (2009)	52/66	49/64	1.14 (0.50–2.60)	0.76
Schreiner exp. (2025)	73/77	66/67	0.28 (0.03–2.54)	0.26

Forest plot of the random-effects meta-analysis comparing diagnostic sufficiency in fasting versus non-fasting patients across three randomized studies (n = 378). **The pooled OR was 0.97 (95% CI 0.51–1.86; p = 0.92).**
Heterogeneity estimates: $\tau^2 = 0.00$, $I^2 = 0\%$, Q = 1.38, df = 2, p = 0.50.

Figure S8a2. Forest plot of overall diagnostic sufficiency (sensitivity with Goldschmidt – aorta only).

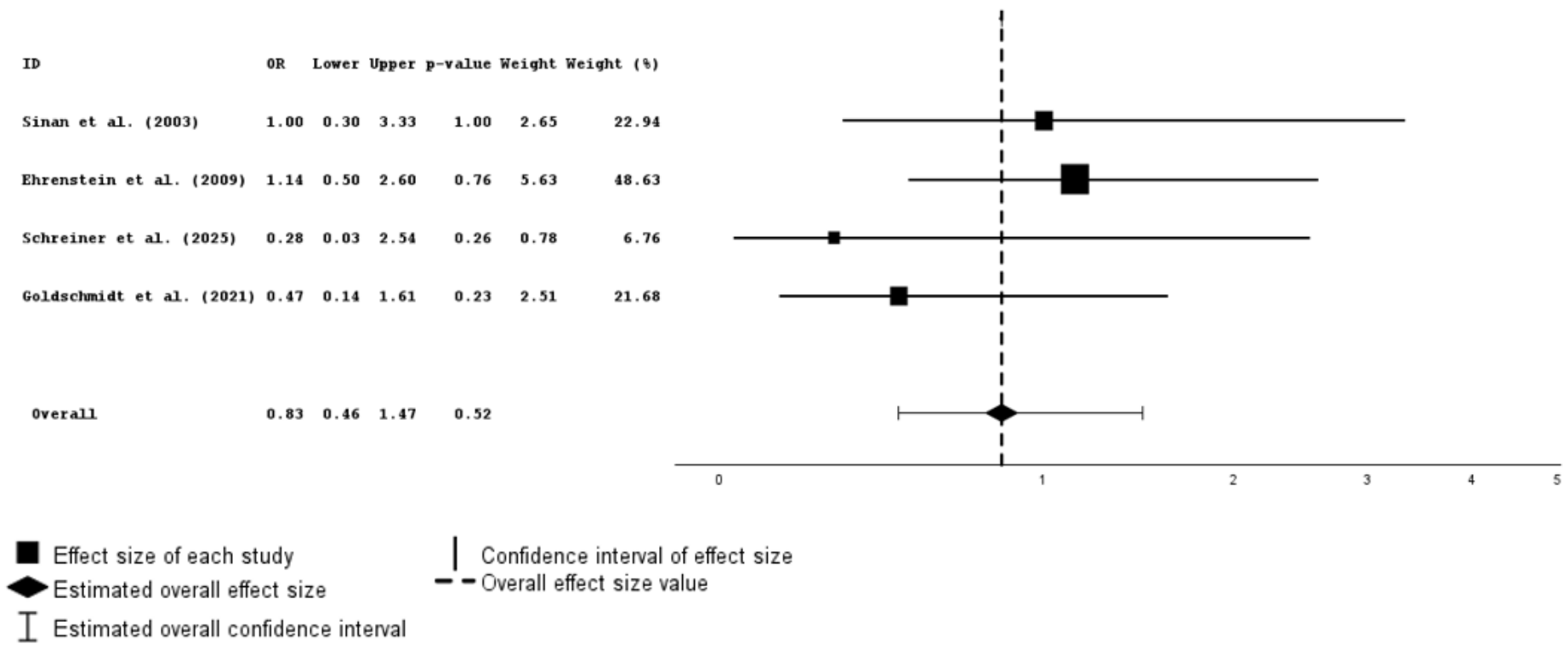

Study (Year)	Fasting (n/N)	Non-fasting (n/N)	OR (95% CI)	p-value
Sinan (2003)	46/52	46/52	1.00 (0.30–3.33)	1.00
Ehrenstein (2009)	52/66	49/64	1.14 (0.50–2.60)	0.76
Schreiner exp. (2025)	73/77	66/67	0.28 (0.03–2.54)	0.26
Goldschmidt (2021)	21/30	25/30	0.47 (0.14–1.61)	0.23

Sensitivity analysis of the random-effects meta-analysis comparing diagnostic sufficiency in fasting versus non-fasting patients, including Goldschmidt (2021) alongside the three randomized trials ($n = 438$). **The pooled OR was 0.83 (95% CI 0.46–1.47; p = 0.52).** Heterogeneity: $\tau^2 = 0.00$, $I^2 = 0\%$, $Q = 2.43$, $df = 3$, $p = 0.49$.

Figure S8a3. Forest plot of overall diagnostic sufficiency (mirror primary analysis with inexperienced examiners).

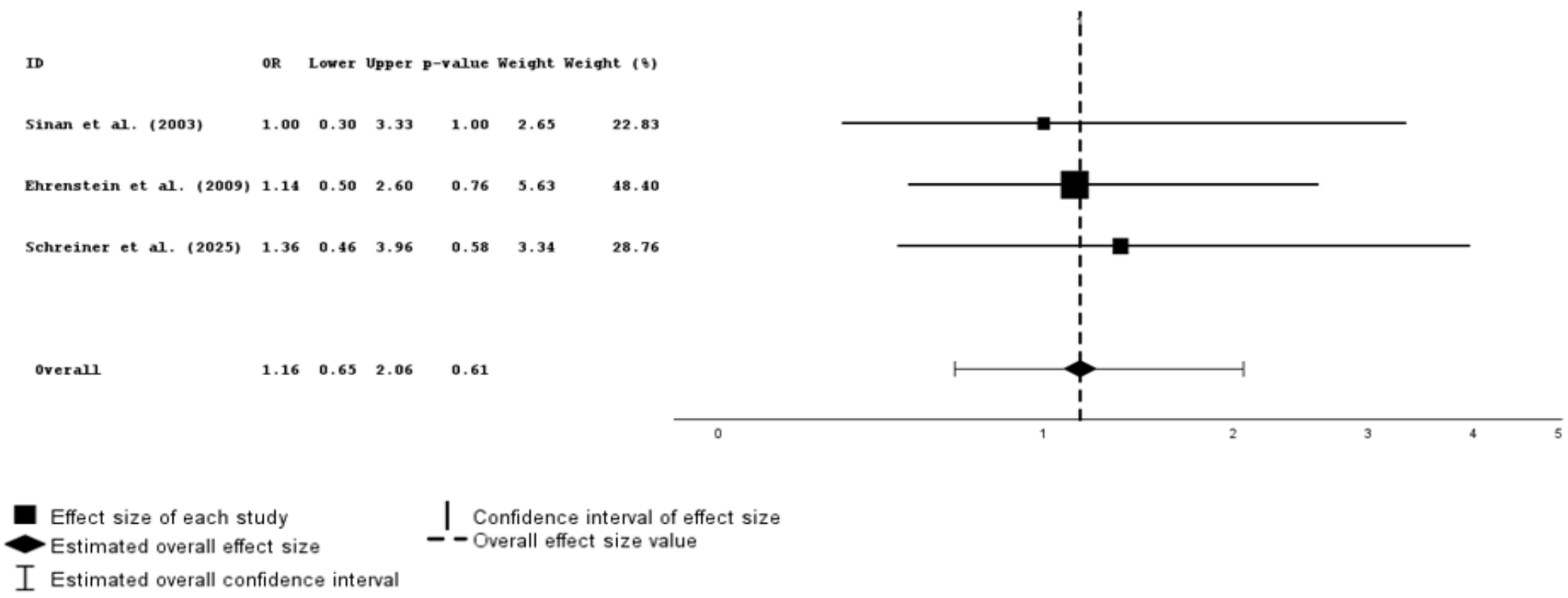

Study (Year)	Fasting (n/N)	Non-fasting (n/N)	OR (95% CI)	p-value
Sinan (2003)	46/52	46/52	1.00 (0.30–3.33)	1.00
Ehrenstein (2009)	52/66	49/64	1.14 (0.50–2.60)	0.76
Schreiner inexp. (2025)	70/77	59/67	1.36 (0.46–3.96)	0.58

Mirror analysis of the random-effects meta-analysis comparing diagnostic sufficiency in fasting versus non-fasting patients across three randomized studies ($n = 378$), experienced examiner dataset replaced by inexperienced examiner dataset from Schreiner et al. (2025). **The pooled OR was 1.16 (95% CI 0.65–2.06; p = 0.61).** Heterogeneity estimates: $\tau^2 = 0.00$, $I^2 = 0\%$, $Q = 0.14$, $df = 2$, $p = 0.93$.

S8b. Gallbladder Evaluability

Definition

Study	Lenient	Strict
Sinan 2003	Minimum: excellent and acceptable visualization	Only excellent visualization
Ehrenstein 2009	Minimum completely and partially evaluable	Only completely evaluable
Schreiner (2025) (exp./inexp)	Minimum: Likert ≥3 (satisfactory, good, very good) image quality	Only Likert 5 (very good) image quality

Table S8b1. Pooled results of gallbladder evaluability under lenient and strict definitions.

Definition	Schreiner dataset	Studies included	n	Pooled OR (95% CI)	p-value	I²
Lenient	Experienced	3 (incl. Sinan)	350	1.35 (0.31–5.92)	0.69	32%
Lenient (excl. Sinan)	Experienced	2	248	1.04 (0.13–8.33)	0.97	61%
Strict	Experienced	3	350	1.89 (1.16–3.07)	**0.01***	0%
**Mirror Analysis**						
Lenient	Inexperienced	3 (incl. Sinan)	350	2.62 (1.01–6.83)	0.05	0%
Lenient (excl. Sinan)	Inexperienced	2	248	2.59 (0.95–7.07)	0.06	0%
Strict	Inexperienced	3	350	1.76 (1.10–2.82)	**0.02***	0%

*Abbreviations: n = number of participants; OR = odds ratio; CI = confidence interval; * p-value <0.05 = significant, I² = heterogeneity;*

Windler et al. (1985) was included only in the CBD sensitivity analysis, as its composite "biliary tract" endpoint primarily reflected ductal visualization and was therefore partially comparable with CBD evaluability. The study did not report gallbladder evaluability as a separate outcome.

Gallbladder evaluability analyses were restricted to participants with an intact gallbladder. Patients with prior cholecystectomy were excluded from the denominator in all studies, resulting in smaller sample sizes compared with the overall diagnostic sufficiency and CBD analyses.

Figure S8b1. Forest plots of gallbladder evaluability under lenient definition

(A) Experienced examiners

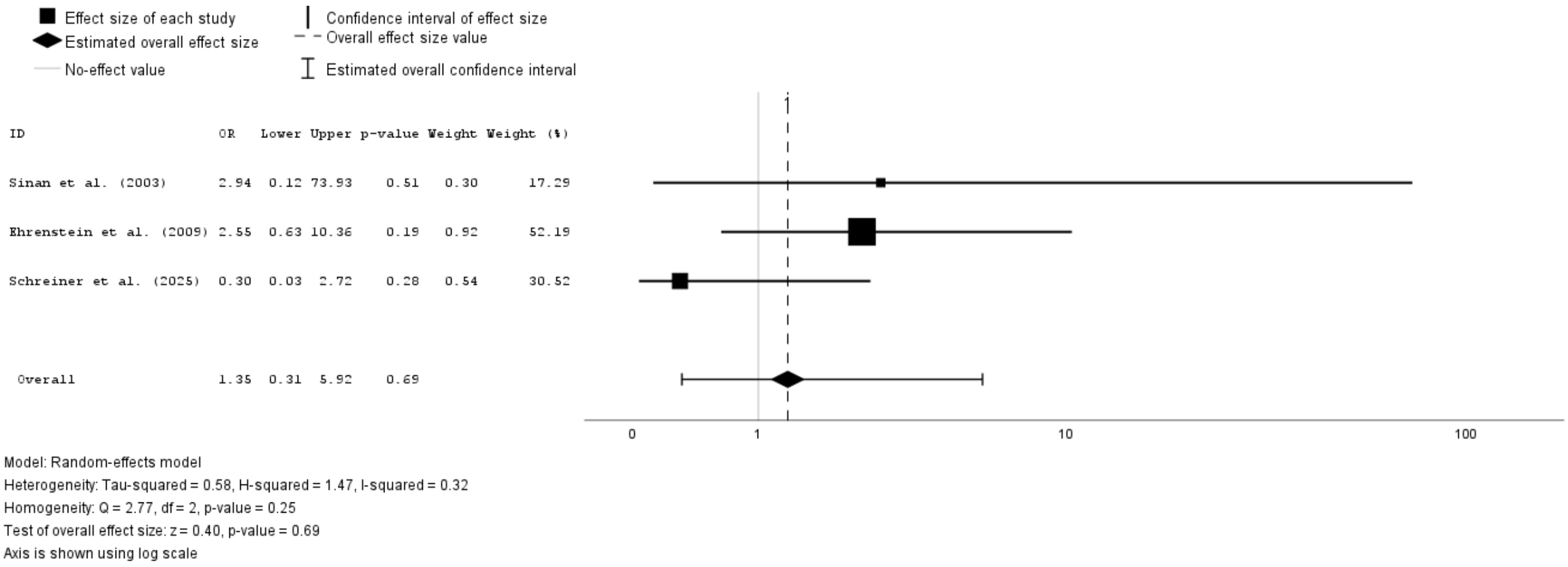

Study (Year)	Fasting (n/N)	Non-fasting (n/N)	OR (95% CI)	p-value
Sinan (2003)	50/50	51/52	2.94 (0.12–73.93)	0.51
Ehrenstein (2009)	59/62	54/61	2.55 (0.63–10.36)	0.19
Schreiner exp. (2025)	65/69	55/56	0.30 (0.03–2.72)	0.28

Random-effects meta-analysis comparing diagnostic image quality for the gallbladder in fasting versus non-fasting patients according to the lenient definition (n = 350). **The pooled OR was 1.35 (95% CI 0.31–5.92; p = 0.69).** Heterogeneity estimates: $\tau^2 = 0.58$, $I^2 = 32\%$, Q = 2.77, df = 2, p = 0.25.

(B) Mirror: Inexperienced examiners

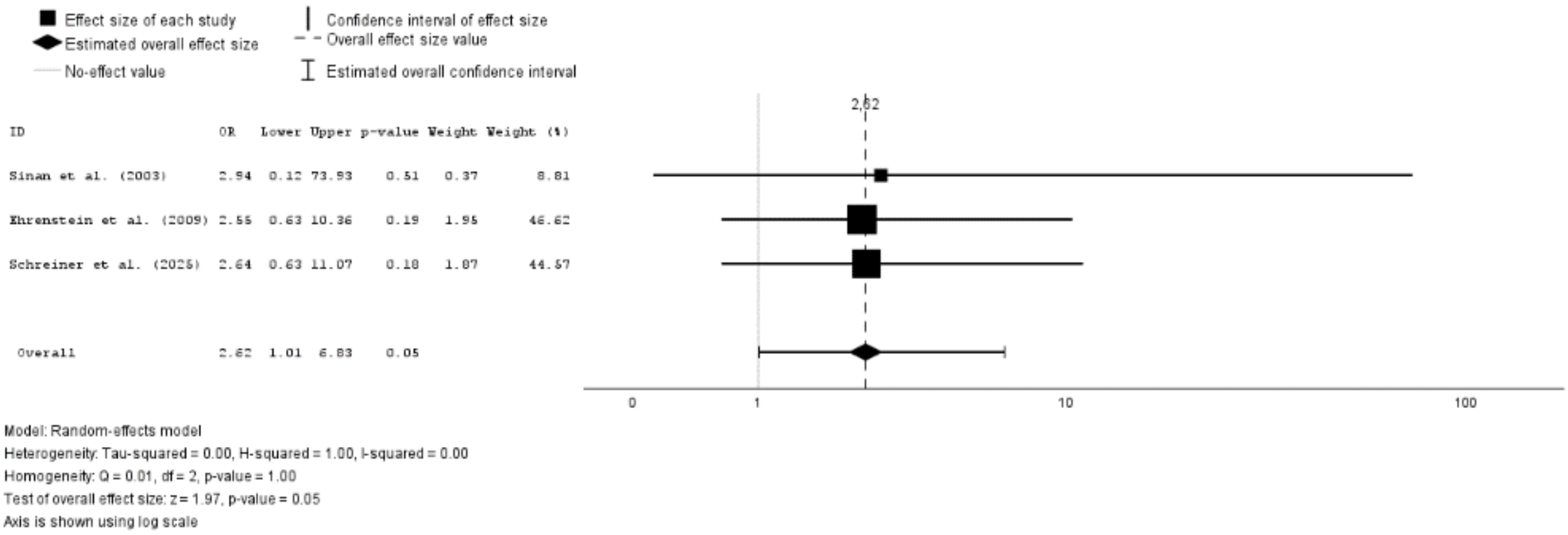

Study (Year)	Fasting (n/N)	Non-fasting (n/N)	OR (95% CI)	p-value
Sinan (2003)	50/50	51/52	2.94 (0.12–73.93)	0.51
Ehrenstein (2009)	59/62	54/61	2.55 (0.63–10.36)	0.19
Schreiner inexp. (2025)	66/69	50/56	2.64 (0.63–11.07)	0.18

Random-effects meta-analysis comparing diagnostic image quality for the gallbladder in fasting versus non-fasting patients according to the lenient definition (n = 350, including the inexperienced examiner dataset from Schreiner 2025). **The pooled OR was 2.62 (95% CI 1.01–6.83; p = 0.05).** Heterogeneity estimates: $\tau^2 = 0.00$, $I^2 = 0\%$, Q = 0.01, df = 2, p = 1.00.

Figure S8b2. Forest plots of gallbladder evaluability under strict definition.

(A) Experienced examiners

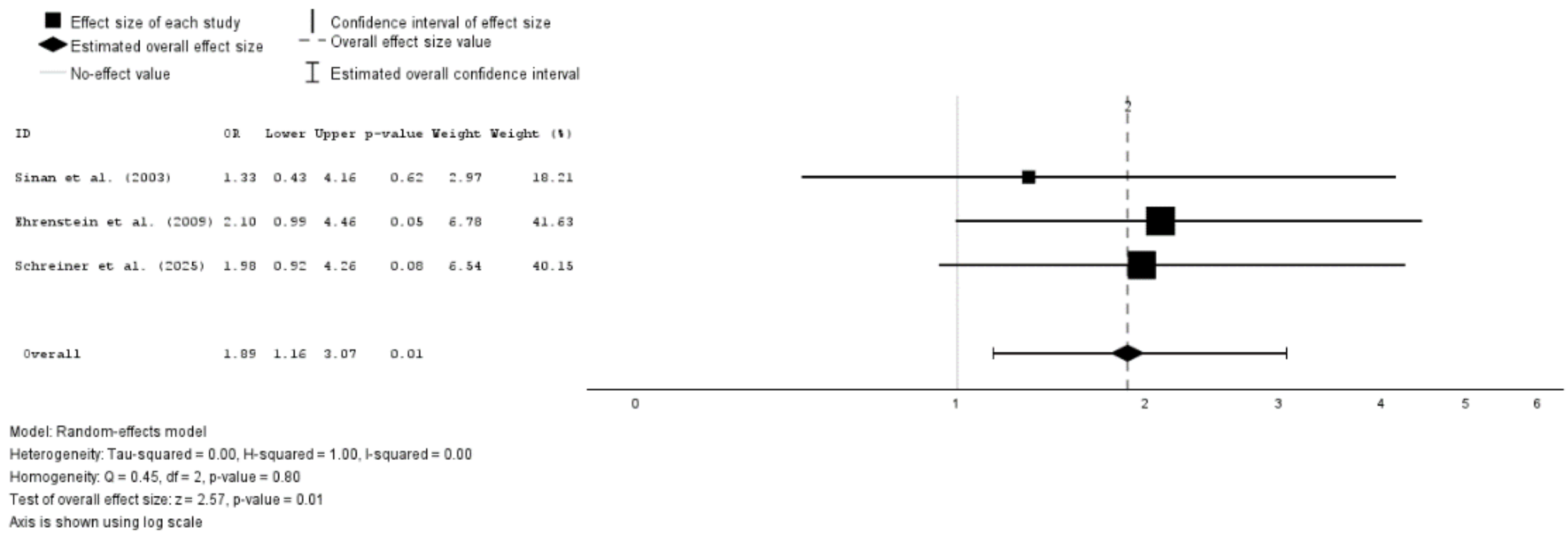

Study (Year)	Fasting (n/N)	Non-fasting (n/N)	OR (95% CI)	p-value
Sinan (2003)	44/50	44/52	1.33 (0.43–4.16)	0.62
Ehrenstein (2009)	45/62	34/61	2.10 (0.99–4.46)	0.05
Schreiner exp. (2025)	52/69	34/56	1.98 (0.92–4.26)	0.08

Random-effects meta-analysis comparing diagnostic image quality for the gallbladder in fasting versus non-fasting patients according to the strict definition (only completely, excellent, or very good visualization) (n = 350). **The pooled OR was 1.89 (95% CI 1.16–3.07; p = 0.01).** Heterogeneity estimates: $\tau^2 = 0.00$, $I^2 = 0\%$, Q = 0.45, df = 2, p = 0.80.

(B) Mirror: Inexperienced examiners

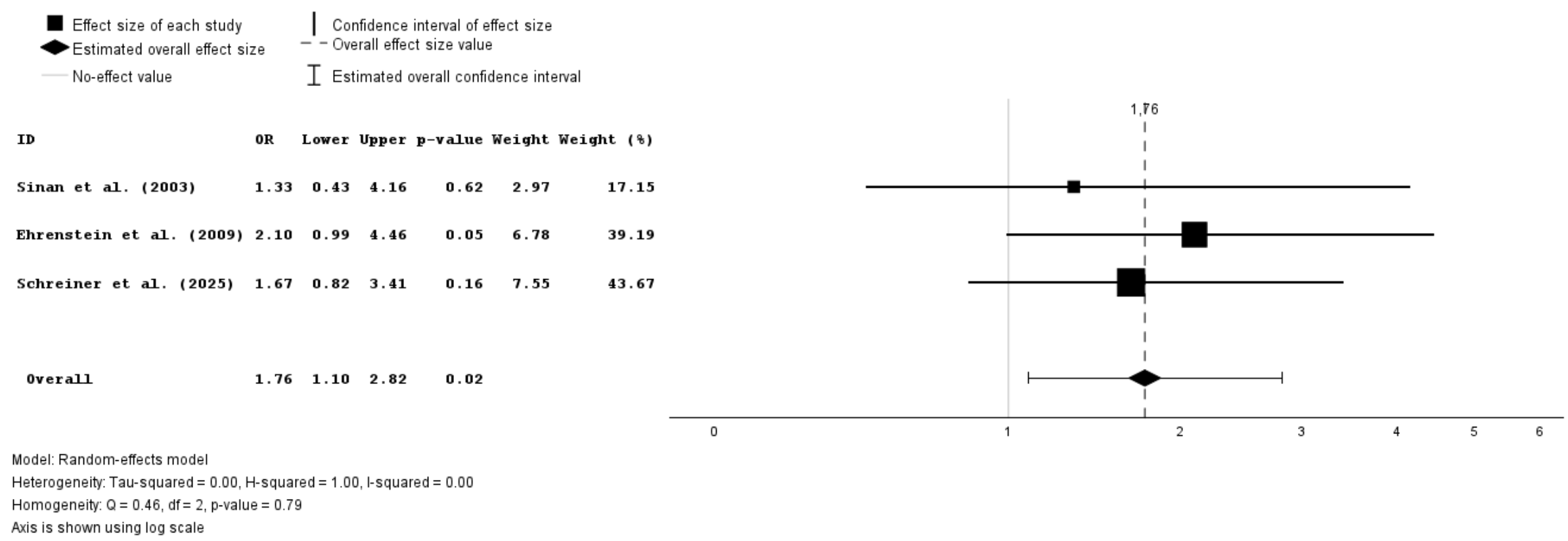

Study (Year)	Fasting (n/N)	Non-fasting (n/N)	OR (95% CI)	p-value
Sinan (2003)	44/50	44/52	1.33 (0.43–4.16)	0.62
Ehrenstein (2009)	45/62	34/61	2.10 (0.99–4.46)	0.05
Schreiner inexp. (2025)	42/69	27/56	1.67 (0.82–3.41)	0.16

Random-effects meta-analysis comparing diagnostic image quality for the gallbladder in fasting versus non-fasting patients according to the strict definition (n = 350, including the inexperienced examiner dataset from Schreiner 2025). **The pooled OR was 1.76 (95% CI 1.10–2.82; p = 0.02).**
Heterogeneity estimates: $\tau^2 = 0.00$, $I^2 = 0\%$, Q = 0.46, df = 2, p = 0.79.

Figure S8b3. Forest plots of gallbladder evaluability (sensitivity analyses)
Sensitivity without Sinan et al., as their study reported a near-ceiling effect of the gallbladder under the lenient definition, which may disproportionately influence the pooled effect.

(A) Experienced examiners (excluding Sinan 2003), lenient definition

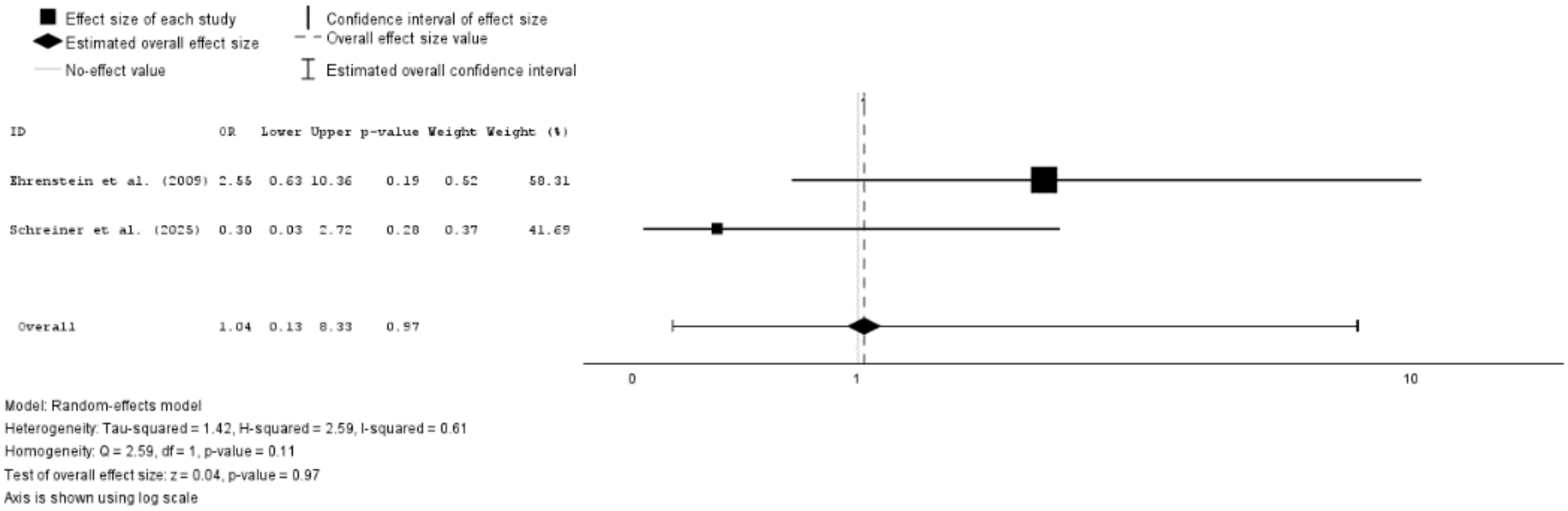

Study (Year)	Fasting (n/N)	Non-fasting (n/N)	OR (95% CI)	p-value
Ehrenstein (2009)	59/62	54/61	2.55 (0.63–10.36)	0.19
Schreiner exp. (2025)	65/69	55/56	0.30 (0.03–2.72)	0.28

Random-effects meta-analysis comparing diagnostic image quality for the gallbladder in fasting versus non-fasting patients according to the lenient definition across two randomized studies (n = 248). The **pooled OR was 1.04 (95% CI 0.13–8.33; p = 0.97).** Heterogeneity estimates: $\tau^2 = 1.42$, $I^2 = 61\%$, Q = 2.59, df = 1, p = 0.11.

(B) Mirror: Inexperienced examiners (excluding Sinan 2003), lenient definition

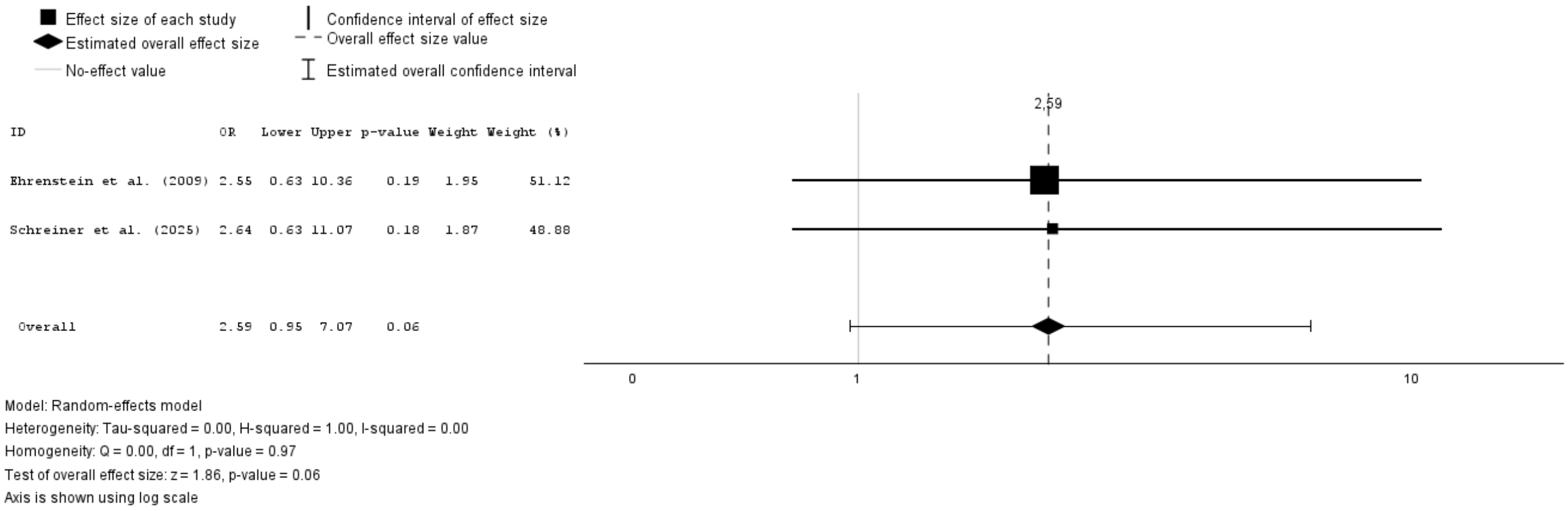

Study (Year)	Fasting (n/N)	Non-fasting (n/N)	OR (95% CI)	p-value
Ehrenstein (2009)	59/62	54/61	2.55 (0.63–10.36)	0.19
Schreiner inexp. (2025)	66/69	50/56	2.64 (0.63–11.07)	0.18

Random-effects meta-analysis comparing diagnostic image quality for the gallbladder in fasting versus non-fasting patients according to the lenient definition across two randomized studies (n = 248, including the inexperienced examiner dataset from Schreiner 2025 and excluding Sinan 2003). **The pooled OR was 2.59 (95% CI 0.95–7.07; p = 0.06).** Heterogeneity estimates: $\tau^2 = 0.00$, $I^2 = 0\%$, Q = 0.00, df = 1, p = 0.97.

S8c. Common Bile Duct (CBD) Evaluability

Definition

Study	Bile duct definition
Sinan (2003)	CBD, segment unspecified
Ehrenstein (2009)	Proximal CBD
Schreiner (2025)	CHD (proximal CD)
Windler (1985)	biliary tract = gallbladder, CBD, prox. biliary duct

CBD evaluability was summarized by prioritizing proximal duct segment data where available.

Study	Lenient	Strict
Sinan (2003)	Minimum: excellent and acceptable visualization	Only excellent visualization
Ehrenstein (2009)	Minimum completely and partially evaluable	Only completely evaluable
Schreiner (2025) (exp./inexp.)	Minimum: Likert ≥3 (satisfactory, good, very good) image quality	Only Likert 5 (very good) image quality
Windler (1985)	Minimum: fair and excellent visibility	Only excellent visibility

Table S8c1. Pooled results of CBD evaluability under lenient and strict definitions.

Definition	Schreiner dataset	Studies included	n	Pooled OR (95% CI)	p	I²
Lenient	Experienced	3	378	3.06 (1.19–7.86)	**0.02***	0%
Lenient	Inexperienced	3	378	2.36 (1.10–5.04)	**0.03***	7%
Strict	Experienced	3	378	1.43 (0.93–2.20)	0.11	0%
Strict	Inexperienced	3	378	1.23 (0.78–1.95)	0.37	0%
**Sensitivity** incl. Windler et al. (1985)						
Lenient	Experienced	4	604	2.53 (1.25–5.10)	**0.01***	0%
Lenient	Inexperienced	4	604	2.21 (1.23–3.96)	**0.01***	0%
Strict	Experienced	4	604	1.48 (1.05–2.08)	**0.02***	0%
Strict	Inexperienced	4	604	1.40 (0.99–1.98)	0.05	0%

*Abbreviations: n = number of participants; OR = odds ratio; CI = confidence interval; * p-value <0.05 = significant, I² = heterogeneity;*

Figure S8c1. Forest plot of CBD evaluability under lenient definition.

(A) Experienced examiners

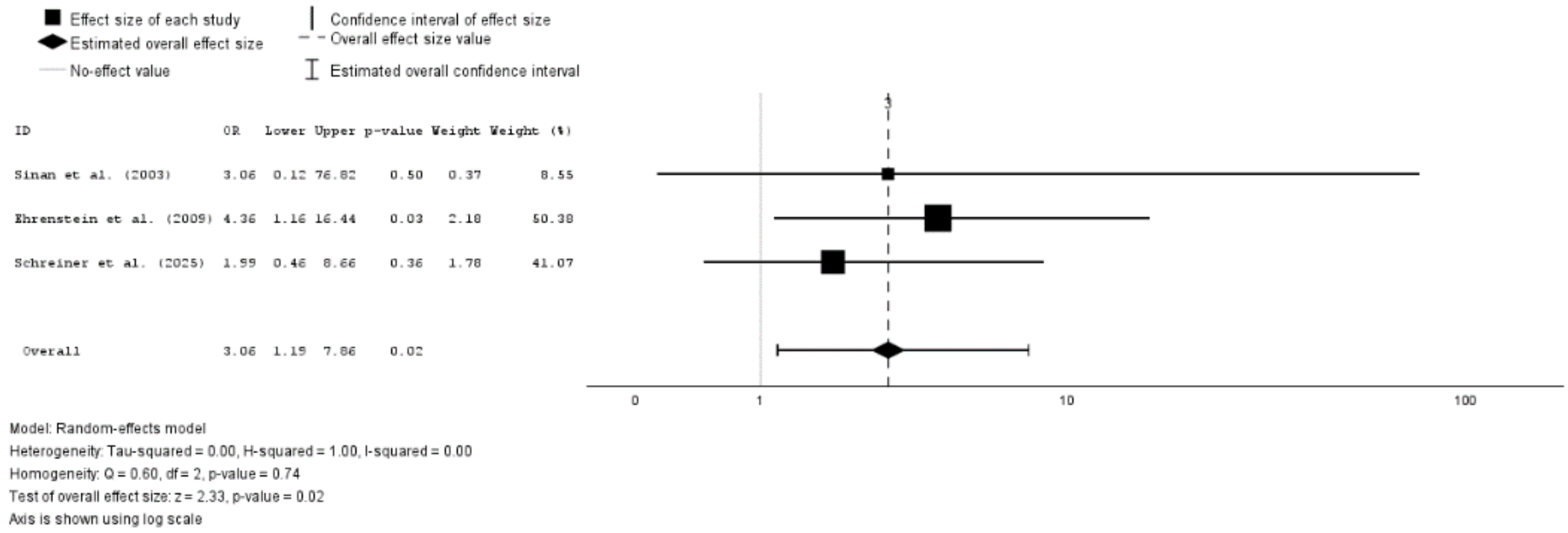

Study (Year)	Fasting (n/N)	Non-fasting (n/N)	OR (95% CI)	p-value
Sinan (2003)	52/52	51/52	3.06 (0.12–76.82)	0.50
Ehrenstein (2009)	63/66	53/64	4.36 (1.16–16.44)	0.03
Schreiner exp. (2025)	74/77	62/67	1.99 (0.46–8.66)	0.36

Random-effects meta-analysis comparing diagnostic image quality for the proximal common bile duct in fasting versus non-fasting patients under the lenient definition (n = 378). **The pooled OR was 3.06 (95% CI 1.19–7.86; p = 0.02),** indicating a significant benefit of fasting. Heterogeneity estimates: τ^2 = 0.00, I^2 = 0%, Q = 0.60, df = 2, p = 0.74.

Excluding Sinan et al. due to a ceiling effect in the fasting arm did not materially alter the pooled effect estimate. Random-effects meta-analysis of two randomized studies (Ehrenstein 2009 and Schreiner 2025; n = 274), excluding Sinan (2003) due to a ceiling effect in the fasting arm, evaluated proximal CBD image quality under the lenient definition and demonstrated that fasting was associated with a significantly higher likelihood of adequate visualization. **The pooled OR was 3.06 (95% CI 1.14–8.21; p = 0.03).** Heterogeneity estimates: τ^2 = 0.00, I^2 = 0 %, Q = 0.60, df = 1, p = 0.44.

(B) Mirror: Inexperienced examiners

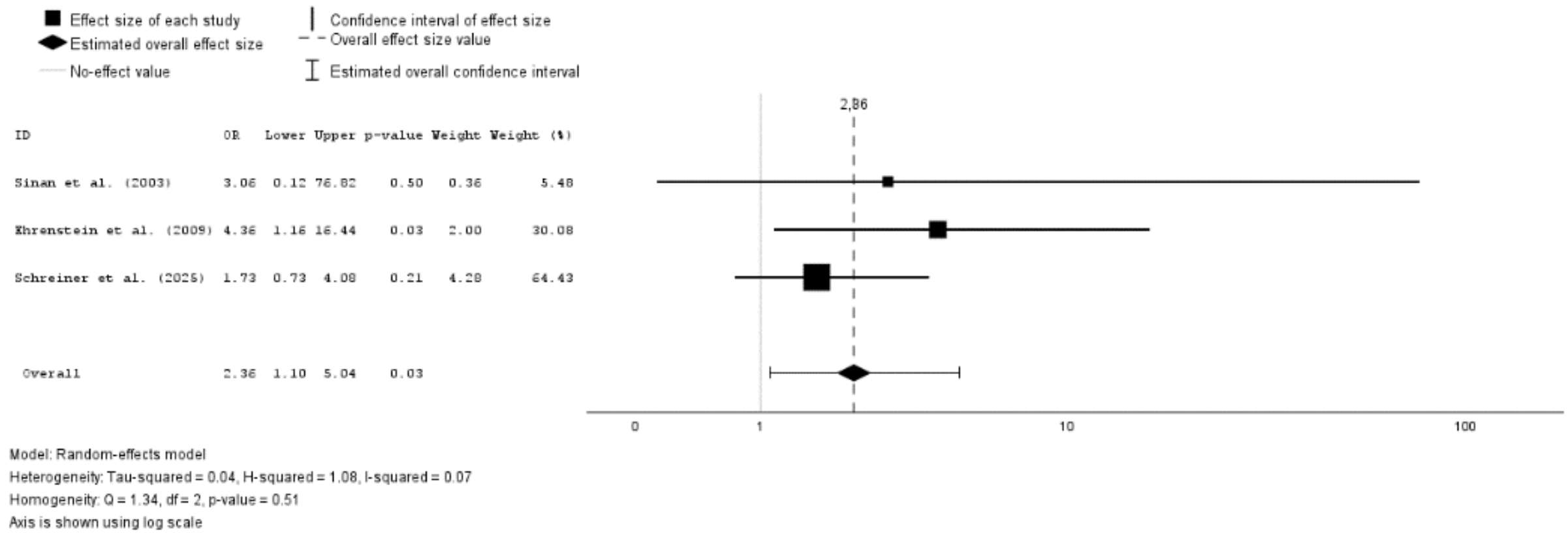

Study (Year)	Fasting (n/N)	Non-fasting (n/N)	OR (95% CI)	p-value
Ehrenstein (2009)	63/66	53/64	4.36 (1.16–16.44)	0.03
Schreiner inexp. (2025)	66/77	52/67	1.73 (0.78–3.86)	0.18
Sinan (2003)	52/52	51/52	3.06 (0.12–76.82)	0.50

Random-effects meta-analysis of three randomized studies (n = 378) under the lenient definition showed that fasting significantly improved proximal CBD evaluability: **pooled OR 2.36 (95% CI 1.10–5.04; p = 0.03**). Heterogeneity estimates: τ^2 = 0.04, I^2 = 7 %, Q = 1.34, df = 2, p = 0.51.

Random-effects meta-analysis of two randomized studies (Ehrenstein 2009 and Schreiner 2025, inexperienced dataset; n = 274) under the lenient definition showed a borderline significant benefit of fasting for proximal CBD evaluability (**pooled OR = 2.38, 95% CI 1.01–5.61; p = 0.05**). Heterogeneity estimates: $\tau^2 = 0.10$, $I^2 = 24$ %, Q = 1.31, df = 1, p = 0.25.

Figure S8c2. Forest plot of CBD evaluability under strict definition.

(A) Experienced examiners

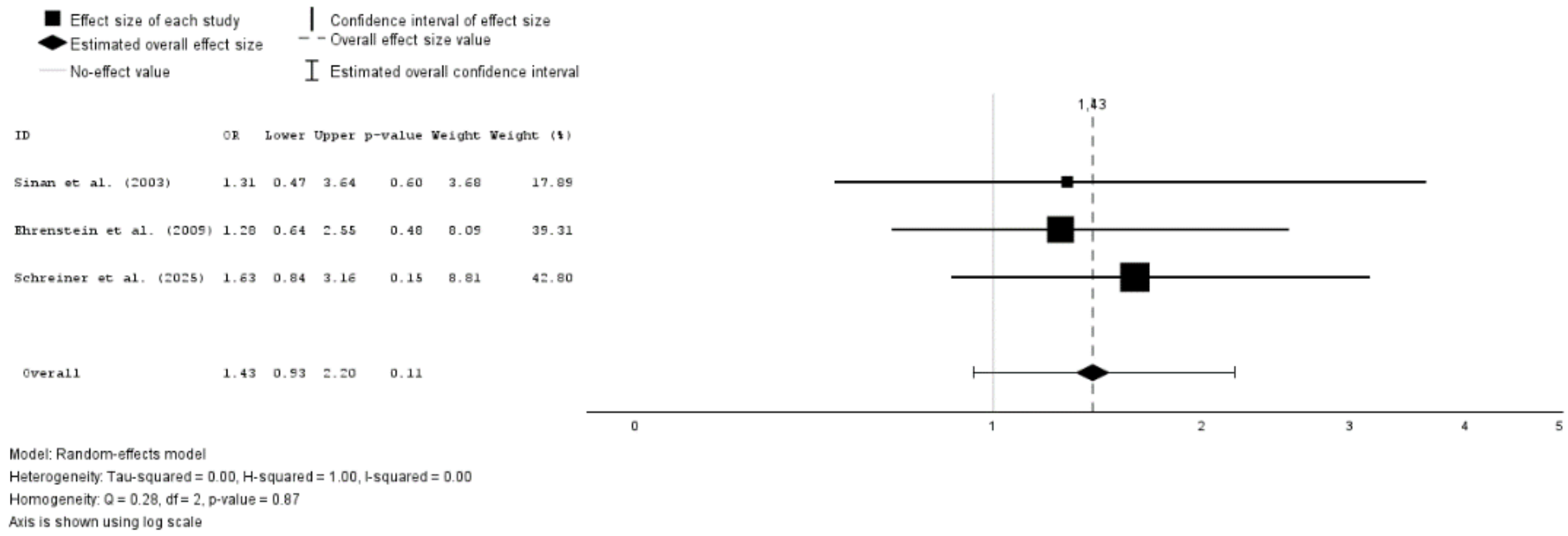

Study (Year)	Fasting (n/N)	Non-fasting (n/N)	OR (95% CI)	p-value
Sinan (2003)	44/52	42/52	1.31 (0.47–3.64)	0.60
Ehrenstein (2009)	35/66	30/64	1.28 (0.64–2.55)	0.48
Schreiner exp. (2025)	45/77	31/67	1.63 (0.84–3.16)	0.15

Random-effects meta-analysis comparing diagnostic image quality for the proximal common bile duct (CBD) in fasting versus non-fasting patients under the strict definition across three randomized studies (n = 378). **The pooled OR was 1.43 (95% CI 0.93–2.20; p = 0.11).** Heterogeneity estimates: $\tau^2 = 0.00$, $I^2 = 0$ %, Q = 0.28, df = 2, p = 0.87.

(B) Mirror: Inexperienced examiners

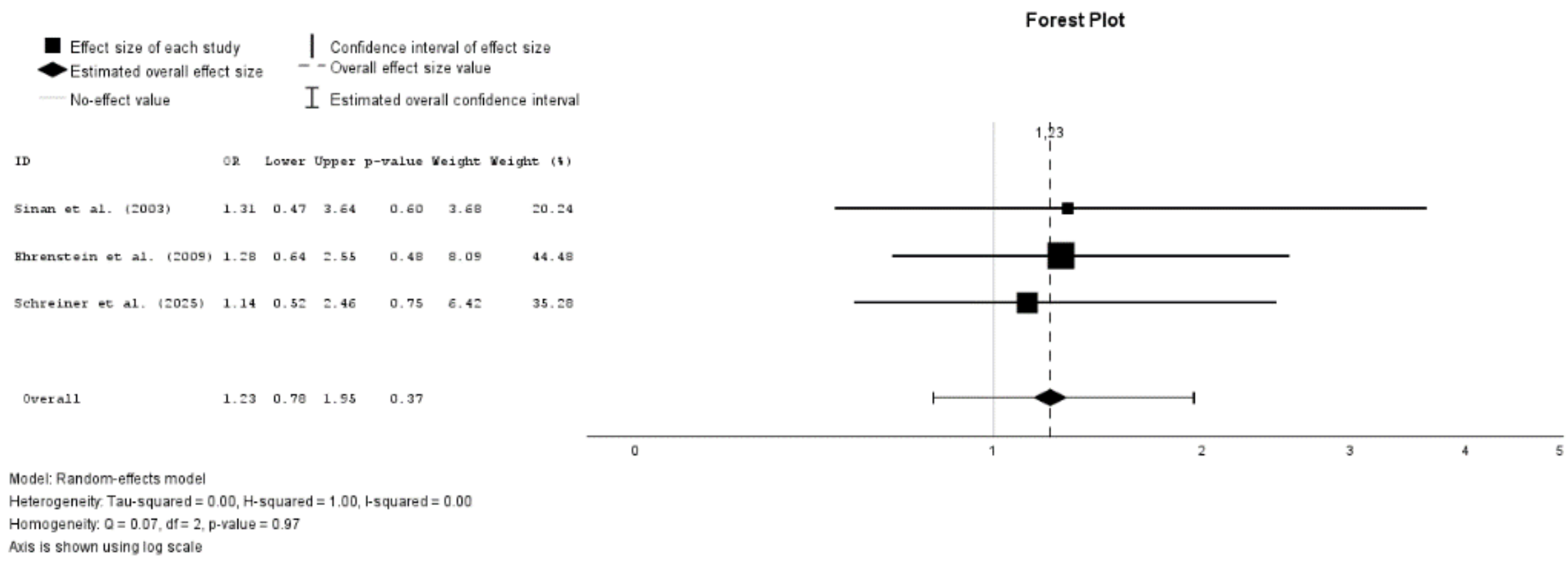

Study (Year)	Fasting (n/N)	Non-fasting (n/N)	OR (95% CI)	p-value
Sinan (2003)	44/52	42/52	1.31 (0.47–3.64)	0.60
Ehrenstein (2009)	35/66	30/64	1.28 (0.64–2.55)	0.48
Schreiner inexp. (2025)	19/77	15/67	1.14 (0.52–2.46)	0.75

Random-effects meta-analysis comparing diagnostic image quality for the proximal common bile duct (CBD) in fasting versus non-fasting patients under the strict definition using inexperienced examiners across three randomized studies (n = 378). **The pooled OR was 1.23 (95% CI 0.78–1.95; p = 0.37).**
Heterogeneity estimates: $\tau^2 = 0.00$, $I^2 = 0$ %, Q = 0.37, df = 2, p = 0.97.

Figure S8c3. Forest plots of CBD evaluability (sensitivity including Windler).

Windler et al. (1985) was included only in the CBD sensitivity analysis, as its composite "biliary tract" endpoint primarily reflected ductal visualization and was therefore partially comparable with CBD evaluability.

(A)Experienced examiners, lenient definition

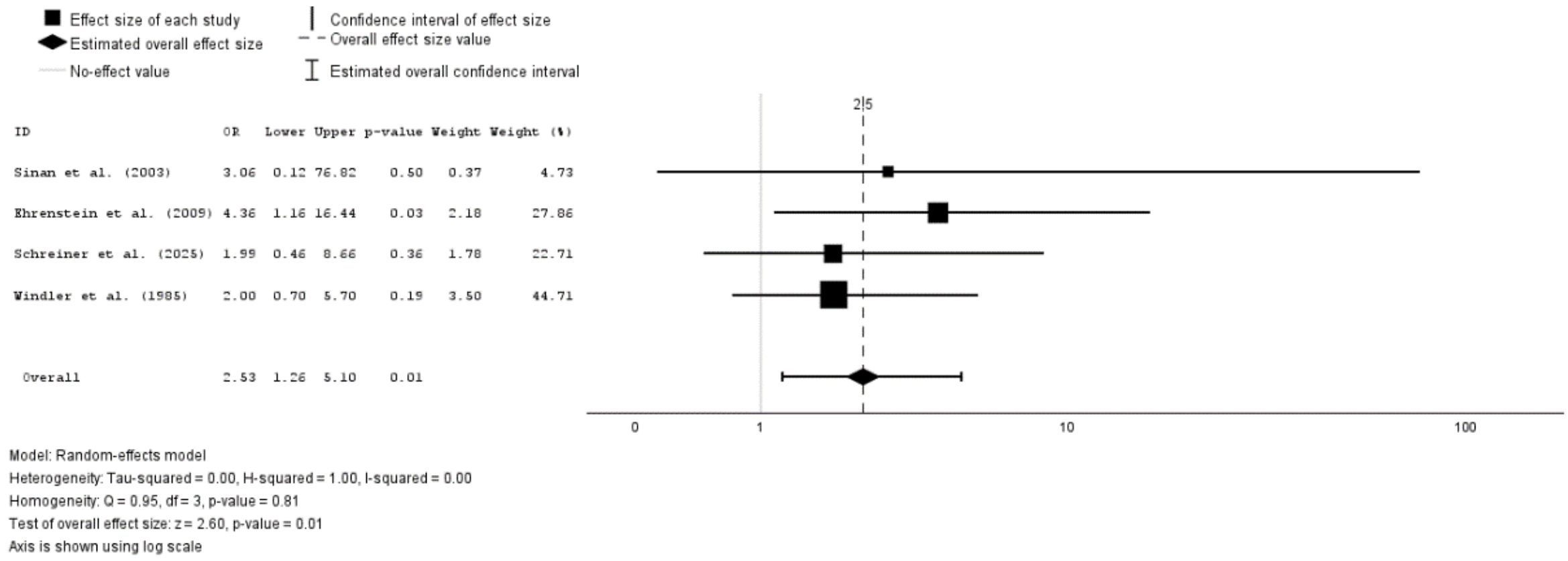

Study (Year)	Fasting (n/N)	Non-fasting (n/N)	OR (95% CI)	p-value
Sinan (2003)	52/52	51/52	3.06 (0.12–76.82)	0.50
Ehrenstein (2009)	63/66	53/64	4.36 (1.16–16.44)	0.03
Schreiner exp. (2025)	74/77	62/67	1.99 (0.46–8.66)	0.36
Windler (1985)	114/121	95/105	2.00 (0.70–5.70)	0.19

Random-effects meta-analysis comparing diagnostic image quality for the proximal common bile duct (CBD) in fasting versus non-fasting patients under the lenient definition, including Windler et al. (1985) (n = 604). **The pooled OR was 2.53 (95% CI 1.25–5.10; p = 0.01).**
Heterogeneity estimates: $\tau^2 = 0.00$, $I^2 = 0$ %, Q = 0.95, df = 3, p = 0.81

(B) Mirror: Inexperienced examiners, lenient definition

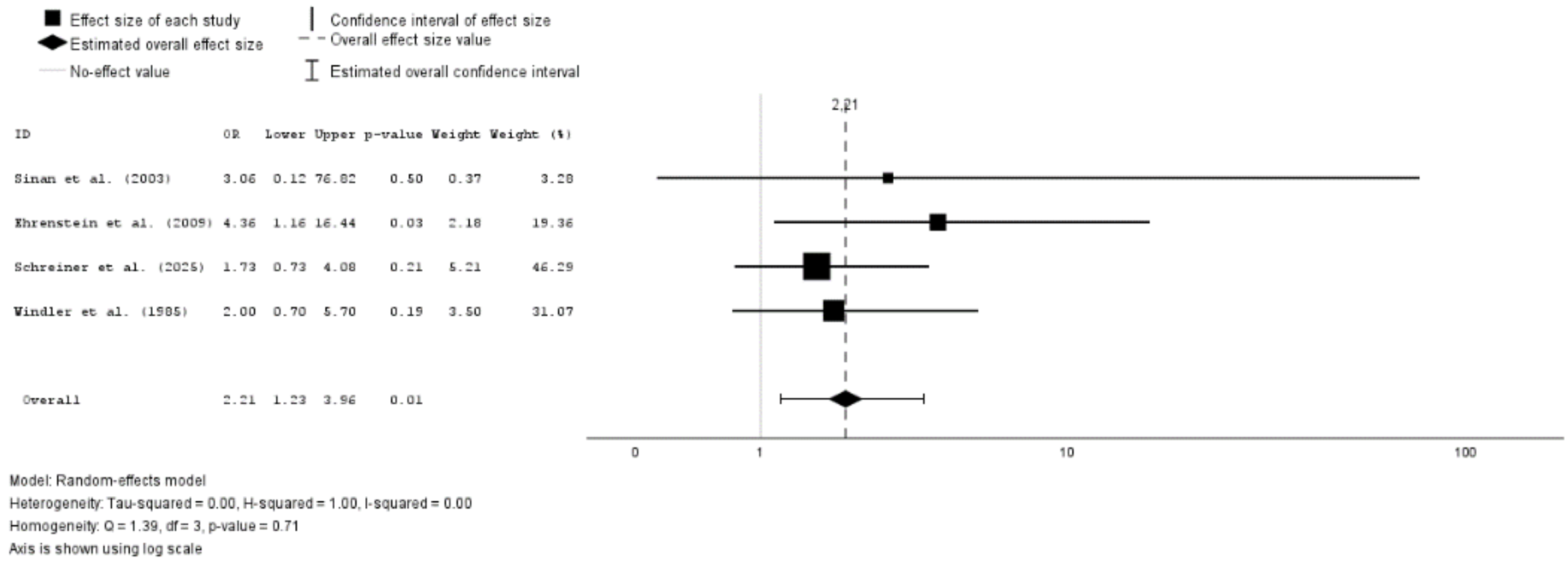

Study (Year)	Fasting (n/N)	Non-fasting (n/N)	OR (95% CI)	p-value
Sinan (2003)	52/52	51/52	3.06 (0.12–76.82)	0.50
Ehrenstein (2009)	63/66	53/64	4.36 (1.16–16.44)	0.03
Schreiner inexp. (2025)	66/77	52/67	1.73 (0.78–3.86)	0.18
Windler (1985)	114/121	95/105	2.00 (0.70–5.70)	0.19

Random-effects meta-analysis comparing diagnostic image quality for the proximal common bile duct (CBD) in fasting versus non-fasting patients under the lenient definition using inexperienced examiners, including Windler et al. (1985) (n = 604). **The pooled OR was 2.21 (95% CI 1.23–3.96; p = 0.01).** Heterogeneity estimates: $\tau^2 = 0.00$, $I^2 = 0$ %, Q = 1.39, df = 3, p = 0.71**.**

(C) Experienced Examiner, strict definition

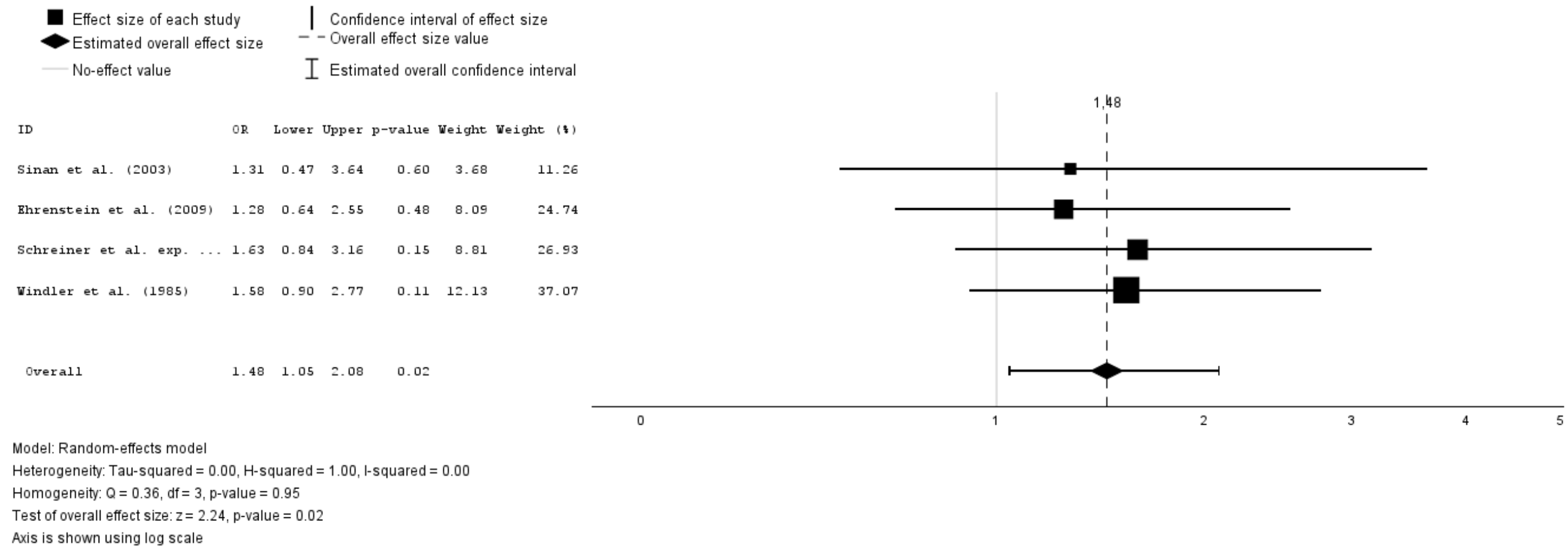

Study (Year)	Fasting (n/N)	Non-fasting (n/N)	OR (95% CI)	p-value
Sinan (2003)	44/52	42/52	1.31 (0.47–3.64)	0.60
Ehrenstein (2009)	35/66	30/64	1.28 (0.64–2.55)	0.48
Schreiner exp. (2025)	45/77	31/67	1.63 (0.84–3.16)	0.15
Windler (1985)	88/121	66/105	1.58 (0.90–2.77)	0.11

Random-effects meta-analysis comparing diagnostic image quality for the proximal common bile duct (CBD) in fasting versus non-fasting patients under the strict definition, including Windler et al. (1985) (n = 604). **The pooled odds ratio (OR) was 1.48 (95% CI 1.05–2.08; p = 0.02).** Heterogeneity estimates: $\tau^2 = 0.00$, $I^2 = 0$ %, Q = 0.36, df = 3, p = 0.95.

(D) Mirror: Inexperienced examiners, strict definition

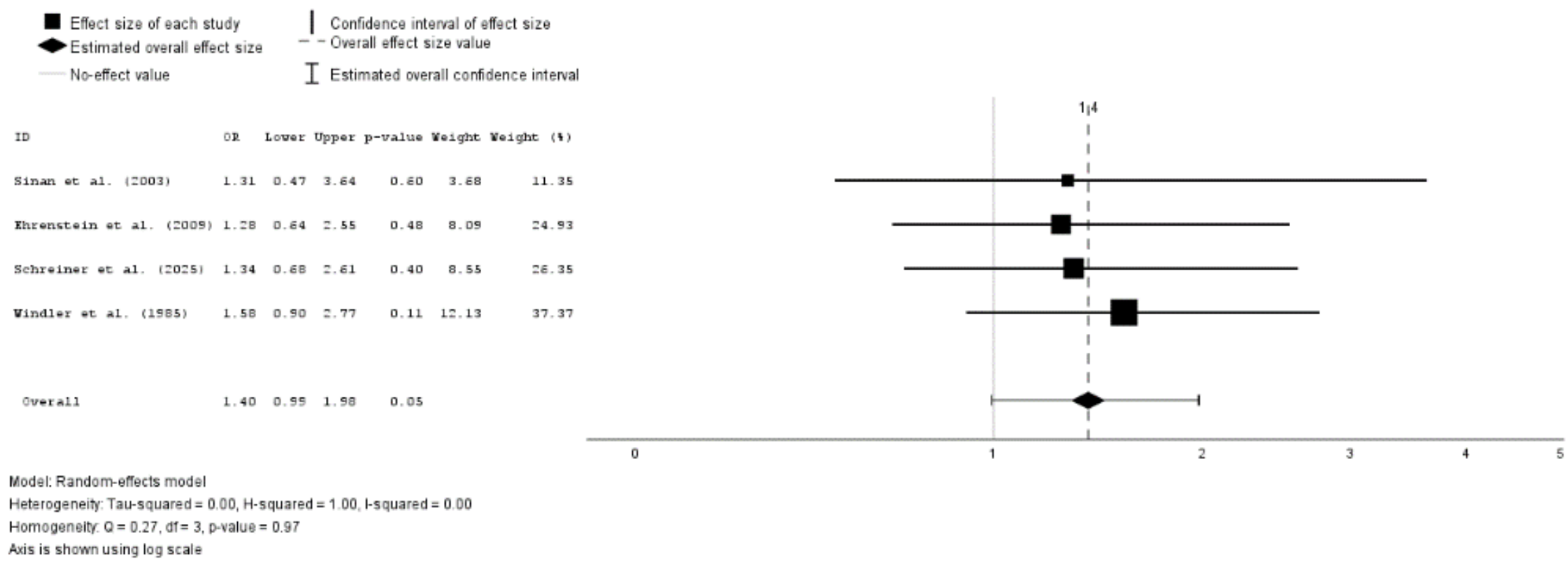

Study (Year)	Fasting (n/N)	Non-fasting (n/N)	OR (95% CI)	p-value
Sinan (2003)	44/52	42/52	1.31 (0.47–3.64)	0.60
Ehrenstein (2009)	35/66	30/64	1.28 (0.64–2.55)	0.48
Schreiner inexp. (2025)	49/77	38/67	1.34 (0.68–2.61)	0.40
Windler (1985)	88/121	66/105	1.58 (0.90–2.77)	0.11

Random-effects meta-analysis comparing diagnostic image quality for the proximal common bile duct (CBD) in fasting versus non-fasting patients under the strict definition using inexperienced examiners, including Windler et al. (1985) (n = 604). **The pooled OR was 1.40 (95% CI 0.99–1.98; p = 0.05).** Heterogeneity estimates: $\tau^2 = 0.00$, $I^2 = 0$ %, Q = 0.44, df = 3, p = 0.93.

Supplement Table S9: GRADE Summary

S9 Table 1: GRADE Summary of Findings:

Diagnostic Sufficiency (Fasting vs. Non-Fasting in Elective Abdominal Ultrasound)

Domain	Action	Assessment
Risk of Bias	No Downgrade	- 1 study truly low risk (Ehrenstein 2009);<br>- Some concerns in 3 studies, mainly due to randomization issues (Goldschmidt 2021, Schreiner 2025, Sinan 2003);<br>- 1 high-risk (Windler 1985) – but this study was only used for sensitivity without influence on results
Inconsistency	Downgrade	- Effect estimates pointed consistently in the same direction across studies.<br>- However, the small number of available trials and wide confidence intervals limit the ability to reliably detect heterogeneity. Although I^2 was 0 percent, the evidence base was considered insufficiently robust to rule out unexplained heterogeneity.
Indirectness	No downgrade	- Population, intervention, comparator, and outcomes closely matched the clinical question.<br>- Studies were conducted in settings and patient groups directly relevant to elective abdominal ultrasound.
Imprecision	Downgrade	- Confidence intervals were wide<br>- small sample size.
Publication Bias	Downgrade	- heterogeneous designs and incomplete publication of results (including conference abstracts without full texts)<br>- several decades of evidence with difference in techniques and publication rules-of-engagement,<br>- Funnel plots were not interpretable due to the small number of studies.
**Overall GRADE**		**Low certainty of evidence**

Supplement S10: Patient-Facing Online Fasting Instructions for Abdominal Ultrasound

S10 Table1: Comparison of Patient-Facing Online Fasting Instructions for Abdominal Ultrasound (Google Search, English, 26th of March 2025)

Website	Country	Broad Fasting Recommended	Fasting Duration Stated	Details on Meal Content	Fluids Allowed	Condition- or Diseases-Specific Instructions[1]	Mentions Guidelines or Evidence
Mayo Clinic, Rochester[2]	USA	No	Not stated	No	Not specified	No	No
Cleveland Clinic, Cleveland[3]	USA	No	Not stated	No	Not specified	No	No
Radiological Society of North America (RSNA)[4]	USA	Conditional	8–12 h (if applicable)	Yes (fat-free)	Yes	No	No
University of Washington Medical Center (UWMC), Seattle[5]	USA	Conditional	6 h (gallbladder only)	No	Clear fluids	No	No
Johns Hopkins Medicine, Baltimore[6]	USA	Yes	8 h	Yes (fat-free)	Yes	No	No
Sansum Clinic, Santa Barbara[7]	USA	Yes	8 h (strict)	No	No	No	No
Mercy Medical Center, Cedar Rapids[8]	USA	Yes	6–8 h	Yes (low-fat)	Yes	No	No
Texas Children's Hospital, Houston[9]	USA	Yes	8 h (≥13 month of age)	No	No	No	No
Wirral University Teaching Hospital, NHS Trust[10]	UK	Yes	6 h	No	Yes	No	No
Whittington Health NHS Trust, London[11]	UK	Yes	6 h	No	Yes	No	No
NHS.uk (National Health Service portal)[12]	UK	No	Deferred to provider	No	Not applicable	No	No
HealthHarmonie, Birmingham[13]	UK	Yes	Not specified	No	Not specified	No	No
Cedars-Sinai Medical Center, Los Angeles[14]	USA	Yes	8 h	No	Yes	No	No

Gateway Diagnostic Imaging, Dallas[15]	USA	Yes	8–12 h	No	Possibly restricted	No	No

[1] Includes any instruction specific to vulnerable patient groups such as those with liver cirrhosis, insulin-dependent diabetes, malnutrition, frailty, or other medical conditions requiring individualized preparation.

[2] https://www.mayoclinic.org/tests-procedures/ultrasound/about/pac-20395177
[3] https://my.clevelandclinic.org/health/diagnostics/4994-abdominal-ultrasound
[4] https://www.radiologyinfo.org/en/info/abdominus
[5] https://www.uwmedicine.org/sites/default/files/2018-10/181019_Radiology_Preps_Ultrasound-Abdominal-Exam.pdf
[6] https://www.hopkinsmedicine.org/health/treatment-tests-and-therapies/abdominal-ultrasound
[7] https://www.sansumclinic.org/medical-services/radiologyGR/ultrasound/ultrasound-of-the-abdomen
[8] https://www.mercycare.org/app/files/public/9c779a4b-f0dc-49bb-addb-54cdaec4f479/US%20Abdomen%20Gallbladder%20Liver%20Aorta.pdf
[9] https://www.texaschildrens.org/content/wellness/preparing-your-child-for-abdominal-ultrasound-how-get-ready-and-what-expect
[10] https://www.wuth.nhs.uk/media/7208/ultrasound-abdomen-patient-information.pdf
[11] https://www.whittington.nhs.uk/default.asp?c=44313
[12] https://www.nhs.uk/conditions/ultrasound-scan/
[13] https://www.healthharmonie.com/post/abdominal-pelvic-ultrasound-scans-preparation-procedure-and-getting-results
[14] https://www.cedars-sinai.org/programs/imaging-center/exams/ultrasound/what-to-expect.html
[15] https://www.gatewaydiagnostic.com/how-do-i-prepare-for-an-abdominal-ultrasound/

S10 Figure 1:

Gemini on 15th of August, 12:05h, not logged in.

Q: Is there an advantage to keep adult patients undergoing a routine elective abdominal ultrasound fasting for any time prior to the examination?

Gemini: Yes, fasting before an abdominal ultrasound can improve image quality by reducing gas and food in the abdomen, which can interfere with the ultrasound waves. However, prolonged fasting can be detrimental, so a moderate fasting period of 6-8 hours is generally recommended.
Sources:
Mayo Clinic, Rajan Singh Sondh, Rajnish Mankotia - Reducing prolonged fasting for abdominal ultrasound scans: BMJ Open Quality 2023; 12:e002396., Whittington Hospital

Here's why fasting is generally recommended:

- **Reduces gas and food in the abdomen:**
  Fasting minimizes the amount of gas and food in the stomach and intestines, which can scatter or absorb ultrasound waves, leading to unclear images.
  Sources: Mayo Clinic, Whittington Hospital

- **Improves gallbladder visualization:**
  A full gallbladder is easier to visualize, and fasting helps keep it filled with fluid.
  Sources:
  I-Med Radiology, Rajan Singh Sondh, Rajnish Mankotia - Reducing prolonged fasting for abdominal ultrasound scans: BMJ Open Quality 2023; 12:e002396.,

- **Enhances visualization of other organs:**
  Clearer images of the liver, pancreas, and other abdominal organs are also achieved through fasting.
  Sources:
  LCDC healthcare, Rajan Singh Sondh, Rajnish Mankotia - Reducing prolonged fasting for abdominal ultrasound scans: BMJ Open Quality 2023; 12:e002396.,

Here's why prolonged fasting can be problematic:

- **Patient discomfort:**
  Prolonged fasting can cause hunger, headaches, and nausea, particularly in patients with diabetes or other conditions.
  Sources:
  Rajan Singh Sondh, Rajnish Mankotia - Reducing prolonged fasting for abdominal ultrasound scans: BMJ Open Quality 2023; 12:e002396., Ehrenstein, B. P., Froh, S., Schlottmann, K., Schölmerich, J., & Schacherer, D. (2009). To eat or not to eat? Effect of fasting prior to abdominal sonography examinations on the

quality of imaging under routine conditions: A randomized, examiner-blinded trial. Scandinavian Journal of Gastroenterology, 44(9), 1048–1054. https://doi.org/10.1080/00365520903075188,
Klein E, Mauri J, Lesserson L-S, Nayyar P. Nil per os duration and anxiety: A single center cross-sectional study. Trends in Anaesthesia and Critical Care. 2023;49:101231.,
Denkyi L. An exploration of pre-operative fasting practices in adult patients having elective surgery. Br J Nurs. 2020 Apr 9;29(7):436-441. doi: 10.12968/bjon.2020.29.7.436. PMID: 32279558.
Ferreira D, Hardy J, Meere W, Butel-Simoes L, McGee M, Whitehead N, Healey P, Ford T, Oldmeadow C, Attia J, Wilsmore B, Collins N, Boyle A. Safety and care of no fasting prior to catheterization laboratory procedures: a non-inferiority randomized control trial protocol (SCOFF trial). Eur Heart J Open. 2023 Oct 17;3(6):oead111. doi: 10.1093/ehjopen/oead111. PMID: 38025651; PMCID: PMC10653665.

- **Increased risk of complications:**
  Prolonged fasting can be dangerous for certain patient groups, such as those with diabetes, who may experience hypoglycemia.
  Sources:
  Rajan Singh Sondh, Rajnish Mankotia - Reducing prolonged fasting for abdominal ultrasound scans: BMJ Open Quality 2023; 12:e002396., Ehrenstein, B. P., Froh, S., Schlottmann, K., Schölmerich, J., & Schacherer, D. (2009). To eat or not to eat? Effect of fasting prior to abdominal sonography examinations on the quality of imaging under routine conditions: A randomized, examiner-blinded trial. Scandinavian Journal of Gastroenterology, 44(9), 1048–1054. https://doi.org/10.1080/00365520903075188,

- **Prolonged diagnosis and management:**
  Inadequate fasting can necessitate rescheduling the ultrasound, leading to delays in diagnosis and treatment.
  Sources:
  Rajan Singh Sondh, Rajnish Mankotia - Reducing prolonged fasting for abdominal ultrasound scans: BMJ Open Quality 2023; 12:e002396.,

In summary: Fasting before an abdominal ultrasound is beneficial for image quality but should be moderate. A **6-8 hour fast is generally recommended** to minimize gas and food interference without causing unnecessary discomfort or risk for the patient.

S10 Figure 2:

ChatGPT-5 on 15th of August, 11:55h. Not logged in.

Q: Is there an advantage to keep adult patients undergoing a routine elective abdominal ultrasound fasting for any time prior to the examination?

ChatGPT-5: Yes, there is an advantage to having adult patients fast before a routine elective abdominal ultrasound. Fasting typically helps improve the quality and accuracy of the ultrasound images. Here's why:

1. **Reduced Gas in the Intestines:** Fasting decreases the amount of gas in the stomach and intestines, which can interfere with the ultrasound waves and obscure the view of abdominal organs.
2. **Better Visualization of the Gallbladder:** The gallbladder is easier to evaluate when the patient has fasted for about 6–8 hours because it remains filled with bile and is not contracted.
3. **Improved Assessment of the Liver, Pancreas, and Other Organs:** Fasting helps reduce bowel content and gas, making it easier to visualize these structures clearly.

So, fasting before an elective abdominal ultrasound is generally recommended to obtain optimal images and improve diagnostic accuracy. Typically, patients are advised to fast **for 6 to 8 hours prior to the exam**.

Q: What about fluid fasting?

ChatGPT-5: Good question! **Fluid fasting**—meaning not drinking any fluids before the ultrasound—is usually **not required** for a routine elective abdominal ultrasound. In fact, patients are often allowed to drink clear fluids like water.
Here's why:

- Drinking water does **not** typically interfere with the ultrasound image quality.
- Staying hydrated can actually help improve the visibility of some structures, like the kidneys and bladder.
- However, patients are generally advised to avoid **milky or opaque liquids** because they can affect the stomach contents and interfere with imaging.

So, for routine abdominal ultrasounds, patients usually fast from **food** for about 6–8 hours but may be allowed to drink clear fluids up until a few hours before the exam, unless otherwise instructed. Always follow specific instructions from the healthcare provider or imaging center.

Q: What if my appointment is at 11:00h?

ChatGPT-5: If your abdominal ultrasound appointment is at 11:00 AM, here's a simple fasting guideline you could follow:

- **Stop eating solid food by around 3:00 AM to 5:00 AM** (6–8 hours before the exam).
- **You can usually drink clear fluids like water up until about 8:00 AM or 9:00 AM**, depending on the instructions you received.
- Avoid any milk, coffee with cream, or opaque drinks after that.

This way, your stomach will be mostly empty, and your gallbladder and other abdominal organs will be in the best state for imaging at 11:00 AM.

If you haven't been given specific instructions, it's a good idea to confirm with your healthcare provider or the imaging center to make sure you follow their protocol. Does that help clarify things?
Q: So I should get up at 3am to eat?

ChatGPT-5: No, you definitely don't need to get up at 3 AM just to eat! The fasting period means you **stop eating by around 3–5 AM**, but you can have your last meal before going to bed the night before.
For example, if your ultrasound is at 11 AM:

- Have your last dinner around 7–9 PM the night before.
- Then fast (no food) overnight until your appointment.
- You can drink water or clear fluids early in the morning if allowed.

So, you won't need to wake up in the middle of the night. Just avoid eating after your evening meal until after the ultrasound. Does that make sense?

Q: So, I basically fast from 7 PM to 11AM? 16 Hours?

ChatGPT-5: Yes, that's basically correct—a **16-hour fast** from 7 PM to 11 AM.
While a 6–8 hour fast is generally recommended, fasting longer than that (like 16 hours) is usually okay for most people and won't harm the quality of the ultrasound. The main thing is to avoid food so your stomach and intestines are empty.
If fasting that long feels difficult or you have medical conditions (like diabetes), it's a good idea to check with your doctor—they might adjust instructions for you.
But for a routine abdominal ultrasound, a 16-hour fast is fine and will give clear images.
Would you like tips on how to handle the fasting or anything else?

Q: I am 75 years old and lost weight already, my BMI is 19 kg/m².

ChatGPT-5: Thanks for sharing that — at 75 years old with a BMI of 19 (which is on the lower side), prolonged fasting might feel more challenging and could affect your energy or well-being.
Here are a few considerations:

- **Talk to your doctor:** Given your age and weight status, it's important to check if the standard fasting instructions are suitable for you. Your doctor might recommend a shorter fasting period or different preparation.
- **Stay hydrated:** Make sure to drink clear fluids like water before the exam unless your doctor says otherwise. Hydration is important to avoid feeling weak or dizzy.
- **Listen to your body:** If you feel unwell during fasting (like dizzy, very weak, or confused), you should contact your healthcare provider immediately.
- **Consider the urgency:** If the ultrasound is routine and not urgent, your doctor might be able to adjust the timing or preparation to make it easier on you.

Would you like me to help draft some questions or points for your doctor regarding this? Or any advice on managing the fasting?

Supplement 11: Professional Societies Guidelines

Institution Year	Guideline	Country	Organ	Fasting requirement	References/<br>references from this SR
**EFSUMB 2016/7**<br><br>European Federation for Ultrasound in Medicine and Biology	EFSUMB Recommendations and Guidelines for Gastrointestinal Ultrasound - Part 1: Examination Techniques and Normal Findings (Long version)	Europe	stomach, small intestine, large intestine, splachnic flow, intestinal motility	**A standard examination of the intestine does not need specific preparation**, LoE 4, GoR B, Strong consensus 12/12<br><br>To **reduce the amount of food and air** in the small bowel a **fasting period of at least 4 hours** is recommended, however, fasting may not significantly improve visibility except in male patients.<br><br>Fasting **>6 hours** is recommended before measuring **splanchnic blood flow**, LoE 4, GoR B, Strong consensus 12/12<br><br>**Overnight fasting** is recommended before assessing **gastrointestinal motility**, LoE 5, GoR C, Strong consensus 12/12	Nylund K, Maconi G, Hollerweger A et al. Ultraschall Med 2017; 38: e1–e15. doi:10.1055/s-0042-115853 (1)<br><br>For the argument that fasting in general ist not necessary Ehrenstein et al. (2) and Sinan et al. (3) are quoted.
**EFSUMB 2018**<br><br>European Federation for Ultrasound in Medicine and Biology	EFSUMB Course Book, 2nd Edition<br><br>Ultrasound of the Liver	Europe	liver	"It is recommended that patients undergo **a period of fasting** […] to maximize the distension of the gall bladder and reduce food residue and gas in the upper gastrointestinal (GI) tract, which may reduce image quality or preclude liver imaging. This is essential for full imaging of the liver and related biliary tree, but may not be required in an acute situation [...]."<br><br>A patient may have **small amounts of still water** by mouth.<br><br>**No smoking for 6-8hours**.	Dietrich CF SC, Jedrzejczyk M. Ultrasound of the liver. In: Dietrich CF, Hrsg. EFSUMB Course Book. 2nd Edition. Aufl. London: EFSUMB; 2018.(4) https://efsumb.org/wp-content/uploads/2023/07/ECB2nd-Chapter02_Liver.pdf<br><br>Ehrenstein et al. (2) is quoted for the period of fasting
**SOR/BMUS 2021**<br><br>Society of Radiographers	Guidelines for Professional Ultrasound Practice. Sixth edition, December 2021.	UK	abdomen, liver, gallbladder, pancreas, biliary tract and others.	The **gallbladder** is best seen with the patient in the left decubitus position and **after a period of fasting (drinking clear fluids only)** to aid distension.	SCoR (Society and College of Radiographers), Ultrasound BBM, Society). Guidelines for professional ultrasound practice. Sixth ed. London: SCoR and BMUS; 2021. (5)

British Medical Ultrasound Society				**Fasting for 4 to 6 hours** to reduce bowel gas improves visibility of the **pancreas**.<br><br>**Fasting** will reduce bowel gas and improve visualization [for visualization of the **bowel**].	https://www.bmus.org/ static/uploads/resources/ 2021_SoR_and_BMUS_guidelines_v1.0_.pdf
**ACR/AIUM 2021**<br><br>American College of Radiology<br><br>American Institute of Ultrasound in Medicine	Practice Parameter for the Performance of an Ultrasound Examination of the Abdomen and/or Retroperitoneum	USA	abdomen, retroperitoneum	Gallbladder and biliary tract:<br>Routine **gallbladder** examination should be conducted on an adequately distended gallbladder whenever possible. In most cases, **fasting for at least 4 hours** prior to elective examination will permit adequate distension of a normally functioning gallbladder. For infants and children, the fasting period should be age appropriate.	AIUM (American Institute of Ultrasound in Medicine). Journal of Ultrasound in Medicine 2022; 41: E1–E8.<br>doi: https://doi.org/10.1002/jum.15874<br>(6)
**JSUM 2021**<br><br>Japan Society of Ultrasonics in Medicine	Manual for abdominal ultrasound in cancer screening and health checkups, revised edition (2021).	Japan	liver, biliary tract, pancreas, spleen, kidneys, and abdominal aorta	Examinees with an examination scheduled for the morning should **not eat solid foods or dairy products after 10 p.m. the night before**.<br><br>Examinees with an examination scheduled for the afternoon **should not eat solid foods or dairy products within 6 h before** the examination.<br><br>Drinking **liquids** (e.g., cold/hot water) to prevent dehydration is allowed up to about **200 ml per time up to 2 h** before the examination	Okaniwa S, Hirai T, Ogawa M et al.). J Med Ultrason (2001) 2023; 50: 5–49.<br>doi:10.1007/s10396-022-01272-w<br>(7)
**ASUM 2018**<br><br>Australasian Society for Ultrasound	Guidelines for Abdominal Ultrasound Examination (Revised February 2018)	Australia, New Zealand and Overseas	abdomen	Any normal arrangements undertaken for patient screening, referral and background profile. The patient has been advised regarding any preparation or special dietary restrictions beforehand.	ASUM (Australasian Society for Ultrasound). Guidelines for abdominal ultrasound examination. Sydney: Australasian Society for Ultrasound in Medicine.; 2018. (8)<br><br>https://www.asum.com.au/ files/public/SoP/curver/Abdo/ Guidelines-for-Abdominal-Ultrasound-Examination.pdf

1. Nylund K, Maconi G, Hollerweger A, Ripolles T, Pallotta N, Higginson A, et al. EFSUMB Recommendations and Guidelines for Gastrointestinal Ultrasound - Part 1: Examination Techniques and Normal Findings (Long version). Ultraschall Med. 2017;38(03):e1–e15.
2. Ehrenstein BP, Froh S, Schlottmann K, Schlmerich J, Schacherer D. To eat or not to eat? Effect of fasting prior to abdominal sonography examinations on the quality of imaging under routine conditions: A randomized, examiner-blinded trial. Scandinavian Journal of Gastroenterology. 2009;44(9).
3. Sinan T, Leven H, Sheikh M. Is fasting a necessary preparation for abdominal ultrasound? BMC Medical Imaging. 2003;3(1).
4. Dietrich CF SC, Jedrzejczyk M. Ultrasound of the liver. In: Dietrich CF, editor. EFSUMB Course Book. 2nd Edition ed. London: EFSUMB; 2018.
5. SCoR (Society and College of Radiographers), Ultrasound BBM, Society). Guidelines for professional ultrasound practice. Sixth ed. London: SCoR and BMUS; 2021.
6. The AIUM Practice Parameter for the Performance of an Ultrasound Examination of the Abdomen and/or Retroperitoneum. Journal of Ultrasound in Medicine. 2022;41(4):E1–E8.
7. Okaniwa S, Hirai T, Ogawa M, Tanaka S, Inui K, Wada T, et al. Manual for abdominal ultrasound in cancer screening and health checkups, revised edition (2021). J Med Ultrason (2001). 2023;50(1):5–49.
8. ASUM (Australasian Society for Ultrasound). Guidelines for abdominal ultrasound examination. . updated 2018 Feb ed. Sydney: Australasian Society for Ultrasound in Medicine.; 2018.

Supplement S12.

Web-based patient-facing preparation recommendations for elective abdominal ultrasound in ambulatory practices and hospitals in the federal state of Berlin, Germany

Methods:

A descriptive web-based assessment of patient-facing fasting recommendations for elective abdominal ultrasound was conducted in the federal state of Berlin, Germany. All acute care hospitals and ambulatory healthcare providers offering abdominal ultrasound were included. Ambulatory providers were identified via the website of the regional Association of Statutory Health Insurance Physicians (Kassenärztliche Vereinigung Berlin; last accessed February 2, 2026) and included all listed radiology and gastroenterology practices, as well as an exemplar sample of more than 30 practices in general internal medicine or primary care. On hospital websites, at least the departments of radiology, gastroenterology, and surgery were screened.

Publicly accessible hospital and practice websites were reviewed for preparation instructions from September 2025 to February 2026. Extracted variables included provider type, recommended fasting duration, and additional preparation advice. Providers without publicly available or explicit fasting information were documented accordingly. Data were extracted manually and summarized descriptively.

Results:

Among 36 acute care hospitals in Berlin, three (8.3%) provided a publicly accessible recommendation issued by their radiology departments. One institution explicitly stated that fasting was not necessary. One recommended an "empty stomach" without further specification. One provided a detailed instruction advising overnight fasting with a light evening meal.

For ambulatory providers, 75 distinct entities were screened, comprising 9 radiology websites covering 25 locations, including two radiology groups accounting for 18 locations, 31 gastroenterology practices, of which 3 were part of multidisciplinary settings, and 35 internal medicine or primary care practices, of which 10 operated within multidisciplinary settings. Four providers (5.3%; three internal medicine/primary care and one gastroenterology practice) had no website.

Information related to abdominal ultrasound was available on 36 of 71 ambulatory websites (50.7%), including all radiology websites, 19 of 30 gastroenterology websites (63.3%), and 8 of 32 internal medicine or primary care websites (25.0%). Of these 36 websites, 21 (58.3%; corresponding to 29.6% of all screened ambulatory websites) explicitly reported patient-facing preparation instructions. These included 4 of 9 radiology websites, one of which represented a radiology group with 10 locations, 12 of 19 gastroenterology websites, and 5 of 8 internal medicine or primary care websites.

Table S12 summarizes patient-facing preparation instructions from the 21 ambulatory practice websites that explicitly provided fasting-related information. Recommendations varied substantially with respect to fasting duration, conditional instructions, and additional restrictions regarding fluid intake, medications, smoking, and dietary composition. Fasting duration was categorized as no fasting, short fasting (3–4 hours), moderate fasting (4–6 hours), or prolonged fasting (≥8 hours or overnight). Prolonged fasting recommendations were most common, reported by 10 of 21 websites (47.6%).

Figure S12. Distribution of patient-facing fasting recommendations among 21 ambulatory practice websites providing preparation instructions for elective abdominal ultrasound in Berlin, Germany.

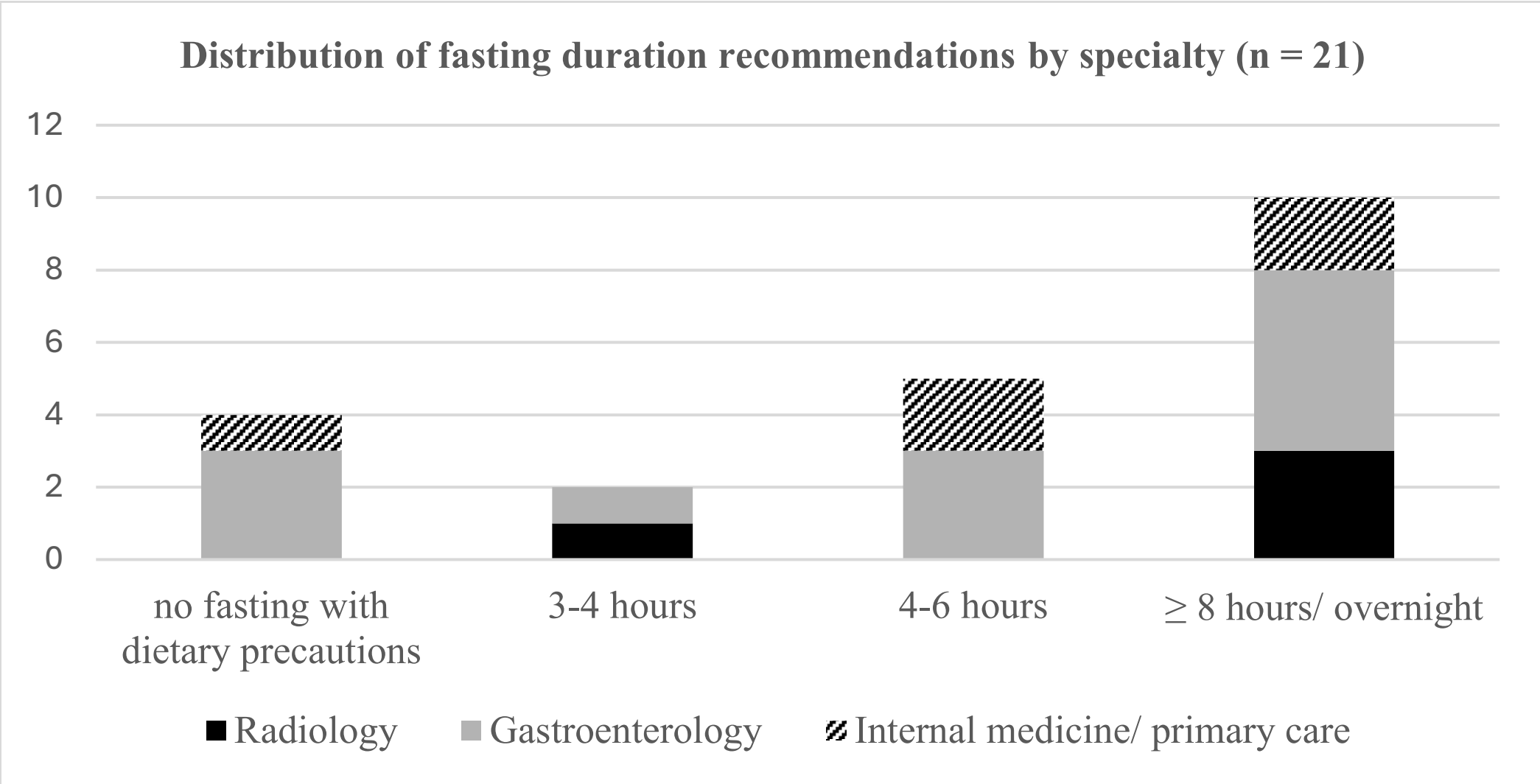

Supplement: Supplementary file 1 [file 11739_2026_4511_MOESM1_ESM.pdf]
